# VPS35 promotes gastric cancer progression through integrin/FAK/SRC signalling-mediated IL-6/STAT3 pathway activation in a YAP-dependent manner

**DOI:** 10.1038/s41388-023-02885-2

**Published:** 2023-11-11

**Authors:** Qingqing Zhou, Feng Qi, Chenfei Zhou, Jun Ji, Jinling Jiang, Chao Wang, Qianfu Zhao, Yangbing Jin, Junwei Wu, Qu Cai, Hua Tian, Jun Zhang

**Affiliations:** 1 Department of Oncology, Ruijin Hospital, Shanghai Jiao Tong University School of Medicine, Shanghai, 200025 China; 2 Shanghai Institute of Digestive Surgery, Ruijin Hospital, Shanghai Jiao Tong University School of Medicine, Shanghai, 200025 China; 3 State Key Laboratory of Oncogenes and Related Genes, Shanghai Cancer Institute, Renji Hospital, Shanghai Jiao Tong University School of Medicine, Shanghai, 200032 China

**Keywords:** Gastric cancer, Oncogenes

## Abstract

VPS35 is a key subunit of the retromer complex responsible for recognising cytosolic retrieval signals in cargo and is involved in neurodegenerative disease and tumour progression. However, the function and molecular mechanism of VPS35 in gastric cancer (GC) remains largely unknown. Here, we demonstrated that VPS35 was significantly upregulated in GC, which was associated with poor survival. VPS35 promoted GC cell proliferation and metastasis both in vitro and in vivo. Mechanistically, VPS35 activated FAK-SRC kinases through integrin-mediated outside-in signalling, leading to the activation of YAP and subsequent IL-6 expression induction in tumour cells. What’s more, combined mass spectrometry analysis of MGC-803 cell and bioinformatic analysis, we found that phosphorylation of VPS35 was enhanced in GC cells, and phosphorylated VPS35 has enhanced interaction with ITGB3. VPS35 interacted with ITGB3 and affected the recycling of ITGB3 in GC cells. Gain- and loss-of-function experiments revealed that VPS35 promoted tumour proliferation and metastasis via the IL-6/STAT3 pathway. Interestingly, we also found that STAT3 directly bound to the VPS35 promoter and increased VPS35 transcription, thereby establishing a positive regulatory feedback loop. In addition, we demonstrated that VPS35 knockdown sensitised GC cells to 5-FU and cisplatin. These findings provide evidence that VPS35 promotes tumour proliferation and metastasis, and highlight the potential of targeting VPS35- and IL-6/STAT3-mediated tumour interactions as promising therapeutic strategies for GC.

## Introduction

Gastric cancer (GC) is the fifth most prevalent cancer globally and represents the third leading cause of cancer-related mortality [1, 2]. The incidence rates of GC vary across different regions of the world. Eastern Asia has the highest age-standardised rate (ASR) for GC incidence at 22.4/100,000 inhabitants, followed by central and eastern Europe (11.3/100,000) and South America (ASR 8.7/100,000). The variations can be attributed to risk factors, genetic features, eating habits and other relevant factors [3]. Despite significant advancements in the understanding and treatment of GC, the prognosis of late-stage GC remains poor. Intratumoural, intrapatient and interpatient heterogeneity in GC is a crucial barrier to drug development for targeted therapies; therefore, it is imperative to identify new predictive biomarkers to improve the survival rate of patients with GC.

We investigated the expression and prognosis in gastric cancer and adjacent normal tissues from Asian and non-Asian GC populations using GEO database and found VPS35 significant upregulation in GC. Bioinformatics analysis showed that there was no significant difference in both the expression of VPS35 and prognosis between Asian and non-Asian GC populations. However, we found elevated VPS35 expression in GC in both Asian and non-Asian GC populations and poor prognosis in patients with high expression of VPS35. Therefore, VPS35 may be a pangenome oncogene. In yeast, the core retromer complex is composed of three proteins: vacuolar protein sorting 35 (VPS35), VPS29 and VPS26. VPS35 functions as the binding factor for membranes of the prevacuolar compartment and is responsible for interactions with the sorting receptors that are to be recycled, suggesting that VPS35 is the key retromer subunit for recognising cytosolic retrieval signals in cargo [4, 5]. Retromer function and dysfunction are linked to neurodegenerative diseases, including Alzheimer’s disease, Parkinson’s disease and even tumours [6–8]. VPS35 has been reported to be significantly associated with the progression and prognosis of several tumours, like breast cancer, and liver cancer [9, 10]. Silencing VPS35 increased N-Ras’s association with cytoplasmic vesicles, diminished GTP loading of Ras, and thus inhibited the proliferation of melanoma cells by inactivating MAPK signalling [11]. VPS35 is defined as an autophagy-related gene because it encodes a retromer subunit that participates in the regulation of autophagy process [12]. Moreover, VPS35 has been implicated in both the activity of the Wnt signalling pathway and the endocytosis process [13, 14]. Previous studies have shown that VPS35 mediated the trafficking of receptors (e.g., IFNAR2, TβRII and FGFR3) and channelled them to different signalling pathways [15]. Moreover, VPS35 knockdown weakened SNX5-induced EGFR degradation in hepatocellular carcinoma (HCC) [16]. Genome-wide functional screening data revealed that VPS35 maybe a potential oncogene in GC [17]. However, the function and molecular mechanism of VPS35 in GC are still not fully understood.

Hippo signalling is an evolutionarily conserved kinase cascade pathway that was originally found to control organ size in *Drosophila melanogaster*, and dysregulation of Hippo signalling contributes to cancer development [18]. Dysregulated signalling by the Hippo pathway has been reported in several cancer types such as breast, liver, prostate and gastric tumours [19–21].To date, several factors have been implicated in the regulation of Hippo signalling, such as cell polarity, contact inhibition, and the actin cytoskeleton [22–24]. However, the molecular drivers and regulators that affect Hippo pathway signalling during cancer development have not been fully characterised.

This study revealed that VPS35 promoted GC cell proliferation and metastasis both in vitro and in vivo. Mechanistically, VPS35 activated FAK-SRC kinases through integrin-mediated outside-in signalling, leading to the activation of YAP and subsequent IL-6 expression induction in tumour cells. VPS35 interacted with ITGB3 and affected the recycling of ITGB3 in GC cells. Gain- and loss-of function experiments revealed that VPS35 promoted tumour proliferation and metastasis via the IL-6/STAT3 pathway. Interestingly, we also found that STAT3 directly bound to the VPS35 promoter and increased VPS35 transcription, thereby establishing a positive regulatory feedback loop. In addition, we demonstrated that VPS35 knockdown sensitised GC cells to 5-FU and cisplatin. These findings provide evidence that VPS35 promotes tumour proliferation and metastasis and highlight the potential of targeting VPS35- and IL-6/STAT3-mediated tumour interactions as promising therapeutic strategies for GC.

## Results

### Upregulated VPS35 expression predicts poor prognosis in GC patients

**Fig. 1:**
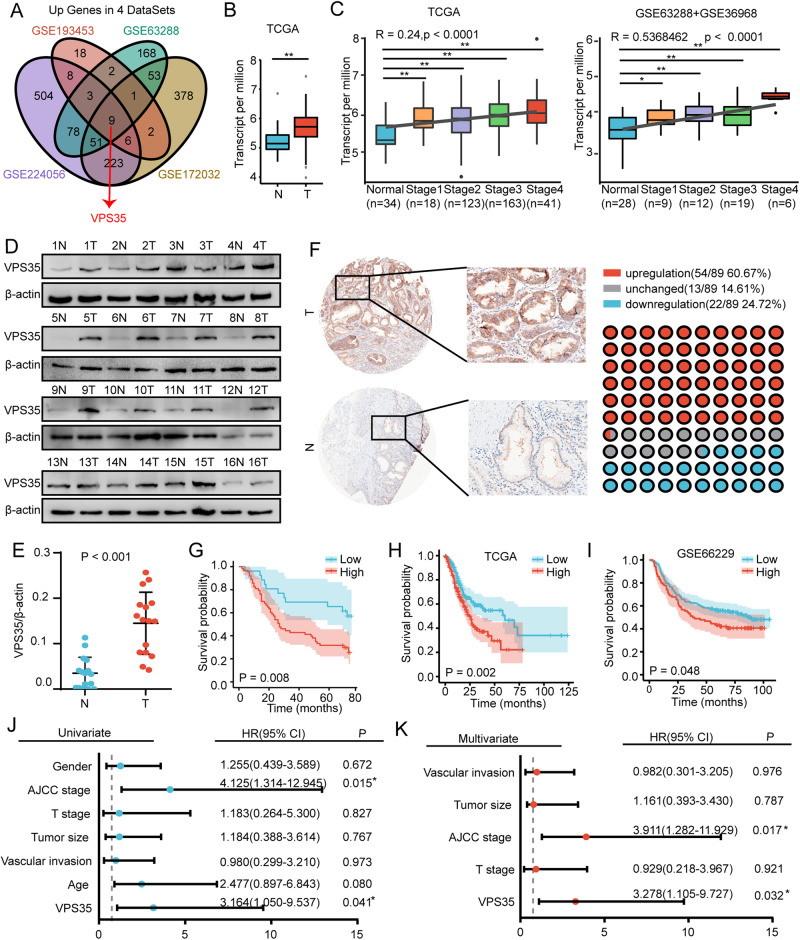
VPS35 is overexpressed and associated with poor prognosis in GC. **A** Venn diagram showing the differential expression of genes in gastric cancer and adjacent normal tissues from GEO datasets (GSE193453, GSE63288, GSE224056 and GSE172032). **B** The expression of VPS35 in GC tissues compared with the corresponding noncancerous gastric tissues using datasets from TCGA. **C** The expression of VPS35 in different GC stages was analysed using datasets from TCGA or GSE63288 + GSE36968. **D** Western blot analysis of VPS35 expression in GC tissues compared with the corresponding noncancerous gastric tissues. **E** Quantification of VPS35 expression in GC tissues compared with the corresponding noncancerous gastric tissues. **F** IHC analysis of VPS35 expression in 89 GC samples. Representative images are shown. **G** Kaplan–Meier survival curves of patients with GC according to VPS35 expression; log-rank test (*n* = 89). **H**, **I** Kaplan–Meier survival curves of patients with GC according to VPS35 expression using datasets from TCGA (*n* = 375) and GEO datasets (*n* = 300); log-rank test. Univariate (**J**) and multivariate (**K**) Cox proportional hazard analyses were conducted to evaluate the HR of VPS35 for overall survival in GC. *p < 0.05, **p < 0.01.

We used four GEO databases to identify differentially expressed genes between GC and adjacent normal tissues, among which nine genes (VPS35, CEACAM6, CLDN4, CXCL1, EREG, HOXA13, LIF, MMP12 and ONECUT2) were upregulated in all four databases, and the most significantly upregulated gene was VPS35 (Fig. 1A). Next, we found that the expression of these nine genes (VPS35, CEACAM6, CLDN4, CXCL1, EREG, HOXA13, LIF, MMP12 and ONECUT2) was significantly higher in GC tissues than in adjacent tissues (Fig. 1B), but only the expression of VPS35 was correlated with the prognosis of GC (Supplementary Fig. 1A). In addition, there was no significant difference in the expression of VPS35 and no significant difference in prognosis between Asian and non-Asian GC populations (Supplementary Fig. 1B). However, we found elevated VPS35 expression in GC in both Asian and non-Asian GC populations and poor prognosis in patients with high expression of VPS35 (Supplementary Fig. 1B). Furthermore, the mRNA level of VPS35 correlated positively with tumour stage in the TCGA and GSE63288 + GSE36968 cohorts (Fig. 1C). Western blotting was also conducted on GC samples from our laboratory, showing that VPS35 expression was elevated in GC tissues compared with noncancerous tissues (Fig. 1D, E). Next, we analysed VPS35 protein expression in patient-derived samples (*n* = 89) and matched normal stomach samples (*n* = 89) using immunohistochemistry and a tissue microarray. Of the 89 pairs, 54 (60.67%) had higher VPS35 protein expression in tumour stomach tissues than in nontumour tissues, and 13 (14.61%) had similar VPS35 expression in both tissue samples, whereas only 22 (24.72%) had lower VPS35 expression in tumour tissues (Fig. 1F). Therefore, these results indicated that VPS35 expression was upregulated in GC.

Considering that VPS35 expression was increased in tumours derived from several different tumours, we next investigated the clinical significance of VPS35 in GC patients. According to the IHC results, patients were divided into low-expression and high-expression groups (Fig. 1F). The results showed that the expression of VPS35 was closely associated with tumour size, T stage, N stage, vascular invasion, and AJCC stage (Table 1). Kaplan‒Meier survival analysis demonstrated that higher levels of VPS35 were associated with shorter overall survival (OS) times (*p* = 0.008; Fig. 1G). Similar results were observed in the TCGA GC cohort and the Gene Expression Omnibus (GEO) datasets (Fig. 1H and I). Moreover, univariate and multivariate COX proportional hazard analyses suggested that a high level of VPS35 was associated with worse survival in GC patients (Fig. 1J, K). Therefore, these findings indicated that VPS35 may be a valuable predictive factor for survival in GC patients.

**Table 1:** Correlation between VPS35 levels in GC patients and their clinicopathological characteristics.

Clinicopathological features	Number	Low expression *N* (%)	High expression *N* (%)	*p* value
Age				
<60	35	11 (31.4)	24 (68.6)	0.857
≥60	54	16 (29.6)	38 (70.4)	
Gender				
Male	61	19 (31.1)	42 (68.9)	0.806
Female	28	8 (28.6)	20 (71.4)	
Tumour size				
≤5 cm	46	21 (45.7)	25 (54.3)	0.001**
>5 cm	43	6 (14.0)	37 (86.0)	
T stage				
I– II	14	9 (64.3)	5 (35.7)	0.003**
III– IV	75	18 (24.0)	57 (76.0)	
N stage				
0–I	37	16 (43.2)	21 (56.8)	0.025^*^
II–III	52	11 (21.2)	41 (78.8)	
Vascular invasion				
Negative	65	24 (36.9)	41 (63.1)	0.026^*^
Positive	24	3 (12.5)	21 (87.5)	
AJCC				
I–II	34	15 (44.1)	19 (55.9)	0.026^*^
III–IV	55	12 (21.8)	43 (78.2)	

^*^p < 0.05

**p < 0.01

### VPS35 promotes GC cell growth in vitro and in vivo

**Fig. 2:**
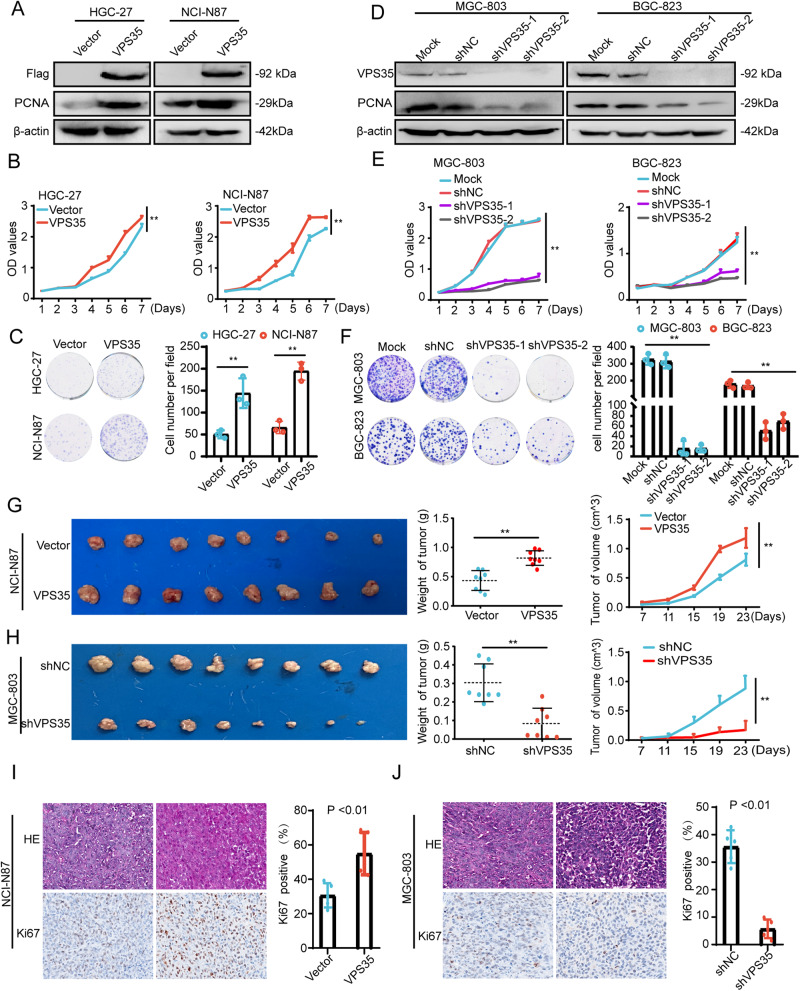
VPS35 increases GC cell proliferation. **A** The expression levels of VPS35 and PCNA were detected by western blotting in GC cells overexpressing VPS35. **B**, **C** The effect of VPS35 overexpression in HGC-27 and NCI-N87 cells on GC cell proliferation was assessed by the CCK8 assay and colony formation assay. **D** The expression levels of VPS35 and PCNA were detected by western blotting in GC cells subjected to VPS35 knockdown. **E**, **F** The effect of VPS35 knockdown in MGC-803 and BGC-823 cells on GC cell proliferation was assessed by the CCK8 assay and colony formation assay. Subcutaneous tumour formation in nude mice (*n* = 8/group). NCI-N87 cells subjected to VPS35 overexpression (**G**) and MGC-803 cells subjected to VPS35 knockdown (**H**) were injected into one flank of each mouse. IHC images of Ki67 expression in xenograft tumours derived from NCI-N87 cells subjected to VPS35 overexpression (**I**) and MGC-803 cells subjected to VPS35 knockdown (**J**). The results for Ki67 positivity (%) are shown in the right panel. *p < 0.05, **p < 0.01.

To verify the role of VPS35 in GC progression, we first observed the expression of VPS35 in GC cell lines by using qRT‒PCR and western blotting (Supplementary Fig. S2A, B). GC cell lines were chosen for gain- or loss-of-function studies according to whether they had low or high endogenous VPS35 levels. The results showed that VPS35 overexpression promoted GC cell proliferation and colony formation ability (Fig. 2A–C and Supplementary Fig. 2C). In contrast, VPS35 knockdown hindered GC cell proliferation and colony formation ability (Fig. 2D–F and Supplementary Fig. 2D). To confirm the in vitro results, we investigated the role of VPS35 in tumour growth using a xenograft model. As shown in Fig. 2G, H, in nude mice, when VPS35 was overexpressed in NCI-N87 cells, the tumours showed enhanced growth. Conversely, tumours derived from MGC-803 cells treated with shVPS35 exhibited smaller volumes and lower weights than tumours derived from MGC-803 cells treated with shNC. Furthermore, the number of Ki67-positive cells was increased in the VPS35-overexpression group and decreased in the shVPS35 group compared with the negative control group (Fig. 2I, J). Overall, these findings suggested that VPS35 promoted GC proliferation.

### VPS35 promotes cell-cycle progression and alters the expression of cell-cycle-related regulators in GC cells

**Fig. 3:**
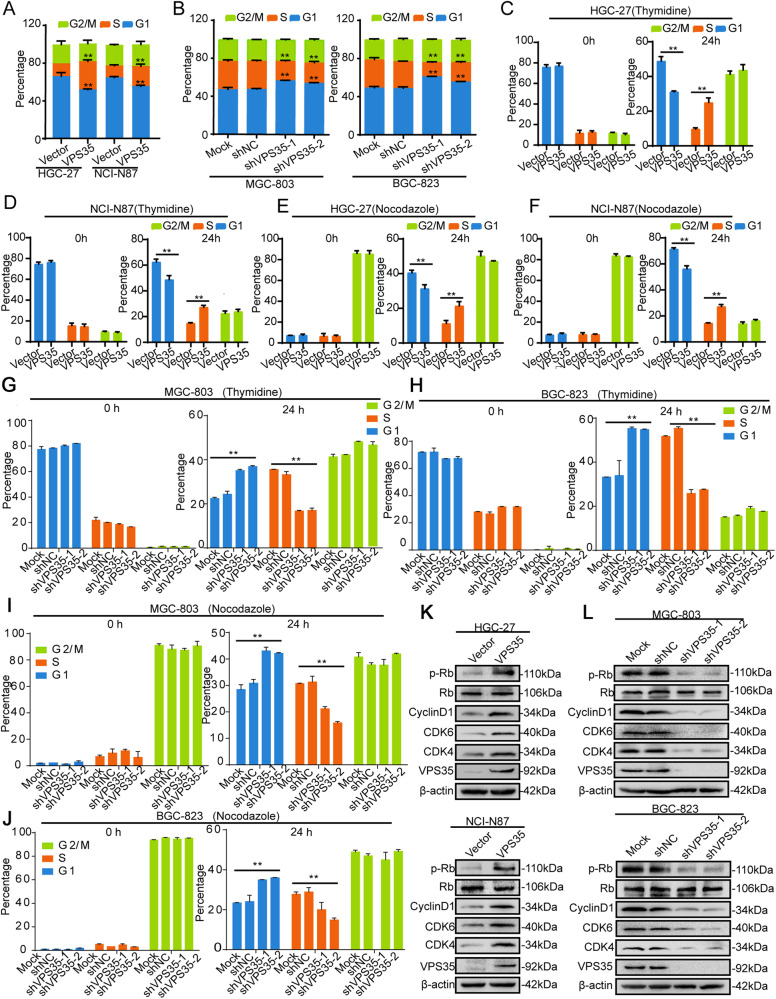
Knockdown of VPS35 induces G1/S phase arrest in GC cells. **A** The cell cycle distribution of VPS35-overexpressing HGC-27 and NCI-N87 cells was analysed by flow cytometry. **B** The cell cycle distribution of MGC-803 and BGC-823 cells subjected to VPS35 knockdown was analysed by flow cytometry. **C**–**F** VPS35-overexpressing cells were collected at 0 and 24 h after release from synchronisation with 2 mmol/L thymidine (**C**, **D**) or 0.8 μM nocodazole (**E**, **F**). **G**–**J** VPS35 knockdown cells were collected at 0 and 24 h after release from synchronisation with 2 mmol/L thymidine (**G**, **H**) or 0.8 μM nocodazole (**I**, **J**). The cell cycle distributions were examined by flow cytometry analysis. **K**, **L** The expression of CDK4/6, cyclin D1, p-Rb, Rb, and VPS35 was detected by Western blotting in VPS35-overexpressing HGC-27 and NCI-N87 cells (**K**) and in MGC-803 and BGC-823 cells subjected to VPS35 knockdown (**L**). *p < 0.05, **p < 0.01.

To further explore the effect of VPS35 on GC cell proliferation, the cell cycle distribution was evaluated by flow cytometry in VPS35-overexpressing and VPS35-knockdown GC cells. As shown in Fig. 3A, B, VPS35 overexpression significantly facilitated the cell cycle transition by increasing the population of S phase cells and decreasing the population of G1 phase cells among HGC-27 and NCI-N87 cells, while VPS35 knockdown in MGC-803 and BGC-823 cells increased the proportion of cells entering the G1 phase and decreased the proportion of cells entering the S phase, indicating that VPS35 knockdown led to G_1_–S arrest. Then, we further added 2 mM thymidine or 0.3 µM nocodazole to synchronise cells at the G_1_–S phase border or G2-M phase border, respectively. At 24 h after release, the results showed that the percentage of cells in S phase was significantly higher in VPS35-overexpressing GC cells than in their corresponding control cells (Fig. 3C–F), while the percentage of cells in S phase was decreased in VPS35-knockdown GC cells compared to their corresponding control cells (Fig. 3G–J). Western blot analysis showed that the expression of related proteins, such as CDK4, CDK6, cyclin D1, and phosphorylated Rb (Ser807/811), was markedly increased in VPS35-overexpressing GC cells and decreased in VPS35-knockdown GC cells versus their control cells (Fig. 3K, L and Supplementary Fig. 3A, B). Taken together, these results illustrated that VPS35 was critical for GC cell cycle progression through the G_1_–S transition.

### VPS35 promotes GC cell migration and invasion

**Fig. 4:**
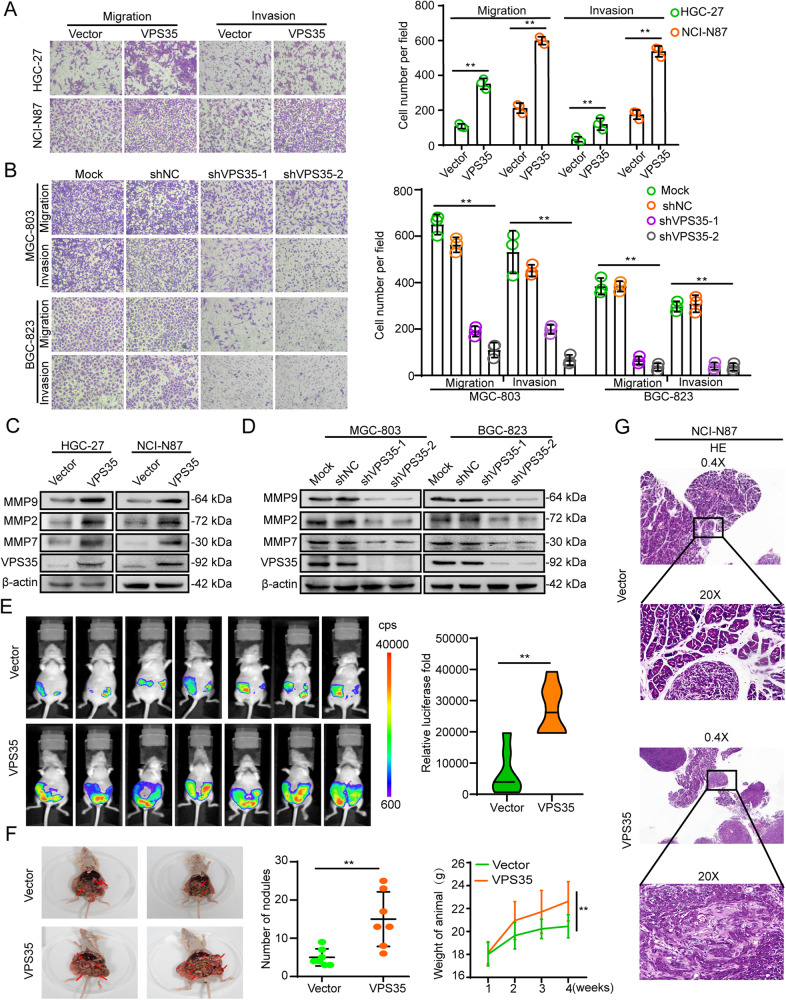
VPS35 increases GC cell metastasis. Transwell assays were performed to assess the effect of VPS35 overexpression (**A**) and VPS35 knockdown (**B**) on GC cell migration and invasion. The expression of MMP9, MMP2, MMP7 and VPS35 was detected by Western blotting in VPS35-overexpressing HGC-27 and NCI-N87 cells (**C**) and in MGC-803 and BGC-823 cells subjected to VPS35 knockdown (**D**). **E**, **F** VPS35 overexpression promoted peritoneal metastasis of gastric cancer cells in vivo. **G** Representative images of peritoneal metastatic nodules formed by NCI-N87-VPS35 and NCI-N87 vector cells. *p < 0.05, **p < 0.01.

Metastasis is an important biological behaviour of malignant tumours. Therefore, we investigated the potential role of VPS35 in GC cell migration and invasion. The results showed that the overexpression of VPS35 promoted GC cell migration and invasion, whereas the knockdown of VPS35 reduced cell migration and invasion (Fig. 4A, B). In addition, western blotting demonstrated that the expression of matrix metalloproteinases (MMP9, MMP2, and MMP7) was upregulated in VPS35-overexpressing GC cells (Fig. 4C and Supplementary Fig. 4A). In contrast, VPS35 knockdown decreased the expression of MMP9, MMP2 and MMP7 (Fig. 4D and Supplementary Fig. 4B). Collectively, these results showed that VPS35 played a crucial role in GC cell metastasis.

We then investigated whether VPS35 could play a vital role in promoting GC cell peritoneal metastasis in vivo by injecting GC cells intraperitoneally into nude mice. As shown in Fig. 4E, F, VPS35 overexpression in NCI-N87 cells resulted in significantly more and larger visible peritoneal nodules than observed in the control group. Moreover, histological examination indicated that the number of peritoneal metastasis nodules was significantly higher and their size was larger in the VPS35-overexpressing group than in the control group (Fig. 4G). Therefore, these results suggested that VPS35 promoted GC cell migration and invasion.

### VPS35 impedes Hippo signalling and activates YAP in GC cells

**Fig. 5:**
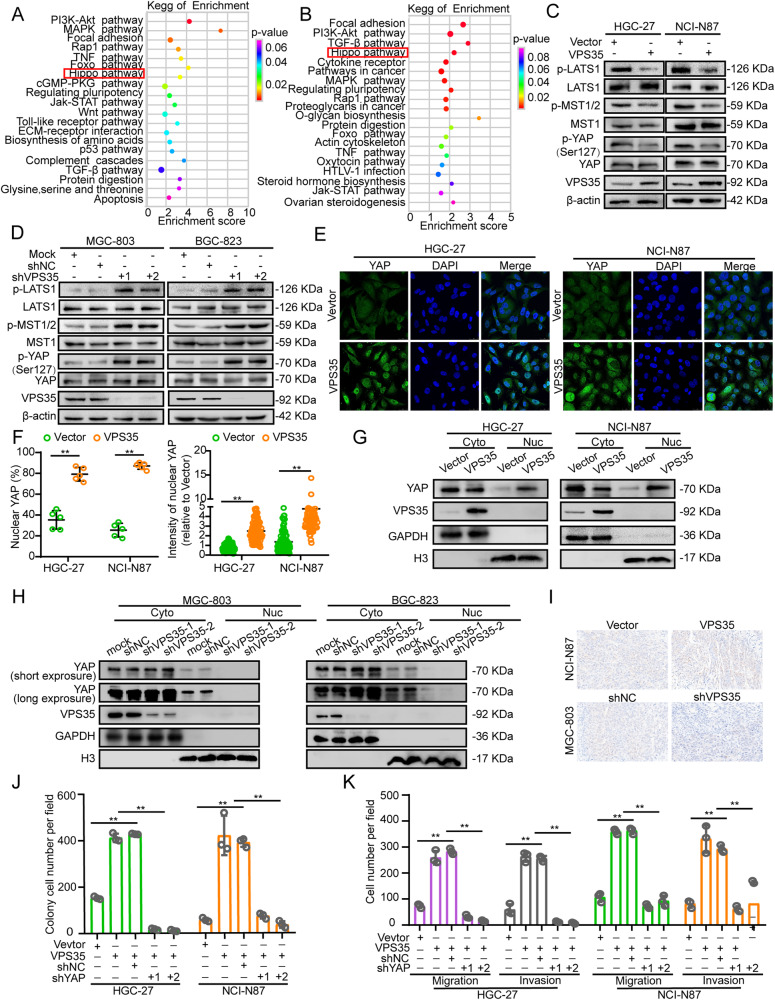
VPS35 impedes Hippo signalling and activates YAP in GC cells. KEGG pathway analysis shows the significantly affected signalling pathways upon VPS35 overexpression in NCI-N87 cells (**A**) and VPS35 knockdown in MGC-803 cells (**B**). **C**, **D** Effects of VPS35 overexpression or knockdown on the protein expression or phosphorylation of Hippo signalling components. **E**, **F** Immunofluorescence assays reveal the subcellular localisation of YAP after VPS35 overexpression (**E**). Quantification of the ratio and the intensity of nuclear YAP staining (**F**). **G**, **H** Nucleocytoplasmic separation and Western blot analysis of YAP cellular localisation changes in VPS35-overexpressing or VPS35-knockdown GC cells. **I** The expression of YAP was detected by immunohistochemical assay in xenograft tumour tissues from NCI-N87-VPS35 and NCI-N87 vector cells or MGC-803 shVPS35 and MGC-803 vector cells. **J** A colony formation assay was performed in VPS35-overexpressing cells treated with YAP shRNA. **K** Cell migration and invasion were analysed in VPS35-overexpressing cells treated with YAP shRNA. *p < 0.05, **p < 0.01.

To investigate the mechanisms of VPS35 in GC cells, total RNAs from VPS35 overexpression or knockdown cells and their corresponding control cells were subjected to RNA sequencing. Kyoto Encyclopedia of Genes and Genomes pathway analysis revealed that Hippo signalling was significantly altered upon VPS35 overexpression and deficiency (Fig. 5A, B). Thus, we speculated that VPS35 could regulate GC proliferation and metastasis via Hippo signalling. Western blotting was performed and demonstrated that VPS35 overexpression in HGC-27 and NCI-N87 cells caused MST1/2 and LATS1 dephosphorylation and that the phosphorylation of YAP at Ser127, an indicator of YAP inactivation, was also decreased (Fig. 5C and Supplementary Fig. 5A). Consistently, VPS35 knockdown activated Hippo signalling in MGC-803 and BGC-823 cells (Fig. 5D and Supplementary Fig. 5B). To further determine whether VPS35 induces YAP activation in GC cells, an immunofluorescence assay was performed and showed that more YAP nuclear translocation occurred in VPS35-overexpressing cells than in vector cells (Fig. 5E, F). In addition, our results showed that the nuclear translocation of YAP was markedly increased in cells upon VPS35 overexpression, wherein a considerable decrease in the levels of nuclear YAP protein was observed upon VPS35 knockdown (Fig. 5G, H). Moreover, immunohistochemical assays also demonstrated that more YAP nuclear translocation occurred in murine xenografts from VPS35-overexpressing NCI-N87 cells, while less YAP nuclear translocation occurred in murine xenografts from VPS35 knockdown cells (Fig. 5I). Collectively, these findings suggested that VPS35 regulated Hippo signalling in GC cells.

To confirm the role of Hippo signalling in VPS35-induced GC proliferation, migration and invasion, we used YAP shRNA to knock down YAP expression in VPS35-overexpressing GC cells (Supplementary Fig. 5C). The results showed that VPS35 overexpression-induced promotion of cell proliferation, migration and invasion was partially reversed by the knockdown of YAP (Fig. 5J, K and Supplementary Fig. 5D–G). Therefore, all of these results indicated that VPS35 promoted GC cell proliferation, migration and invasion through the activation of YAP in a Hippo pathway-dependent manner.

### Integrin-FAK-SRC axis mediates VPS35-induced YAP activation in GC cells

**Fig. 6:**
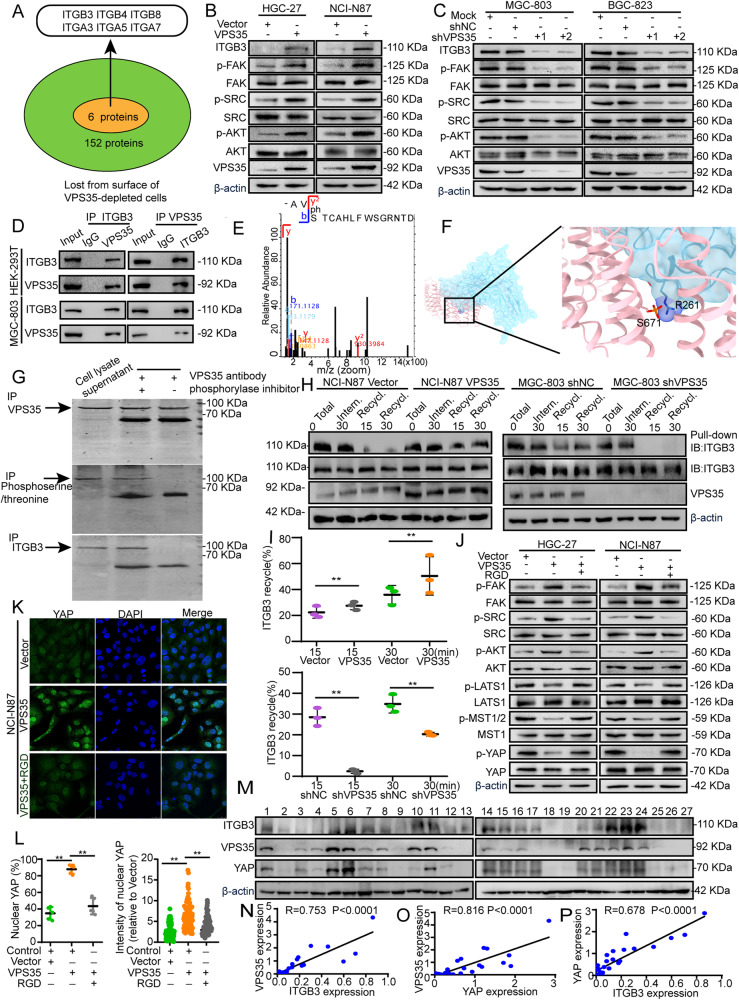
The integrin-FAK-SRC axis mediates VPS35-induced YAP activation in tumour cells. **A** Venn diagram showing the proteins lost from the surface of VPS35-depleted cells. The expression of ITGB3, p-FAK, FAK, p-SRC, SRC, p-AKT and AKT was assessed by Western blotting in VPS35-overexpressing GC cells (**B**) and VPS35-knockdown GC cells (**C**). **D** Endogenous VPS35 was immunoprecipitated with an anti-ITGB3 antibody, with IgG as the negative control, and immunocomplexes were analysed via WB with anti-ITGB3 and anti-VPS35 antibodies. **E** Mass spectrogram of VPS35 phosphorylation. **F** Binding motif between VPS35 and ITGB3. **G** Endogenous ITGB3 was immunoprecipitated with an anti-VPS35 antibody with or without phosphorylase inhibitor, with cell lysate supernatant as the positive control, and immunocomplexes were analysed via WB with anti-ITGB3, anti-phosphoserine/threonine and anti-VPS35 antibodies. **H** VPS35-overexpressing or VPS35-knockdown cells and their corresponding control cells were surface-labelled with cleavable biotin at 4 °C, left alone to allow internalisation or recycling, and examined by Western blotting. **I** Quantification of recycled ITGB3 in VPS35-overexpressing or VPS35-knockdown cells and the corresponding control cells. **J** VPS35-overexpressing GC cells were treated with RGD as indicated, and the levels of p-FAK, FAK, p-AKT, AKT, p-LATS1, LATS1, p-MST1/2, MST1, p-YAP (ser127A) and YAP were determined by Western blotting. Immunofluorescence assay evaluating the nuclear translocation of YAP in VPS35-overexpressing NCI-NN87 cells *with or* without RGD treatment (**K**). Quantification of the ratio and the intensity of nuclear YAP staining (**L**). **M**–**P** Western blot analysis was performed to evaluate the correlations among ITGB3, VPS35, and YAP in GC tissues (*n* = 27). *p < 0.05, **p < 0.01.

Next, we explored the molecular mechanisms by which VPS35 induces YAP activation in tumour cells. YAP can be directly phosphorylated and activated by active SRC in intestine epithelial cells and breast cancer cells [25, 26]. The surface levels of a total of 152 membrane proteins were decreased by more than 1.4-fold in VPS35-depleted cells compared with control cells [27]. Six membrane proteins (ITGB3, ITGB4, ITGB8, ITGA3, ITGA5 and ITGA7) belong to the integrin family (Fig. 6A). ITGB3 plays an important role in the interaction with and activation of focal adhesion kinase [28]. ITGB3 has been proposed to be critical for cancer metastasis, and related drugs are in clinical trials. Thus, we proposed that integrin-FAK-SRC signalling may be involved in VPS35-induced YAP activation in GC. The results showed that VPS35 overexpression or knockdown increased or decreased the levels of ITGB3, phosphorylated FAK, SRC and AKT, *respectively*, in GC cells (Fig. 6B, C and Supplementary Fig. 6A, B). Given the role of the retromer complex in mediating the retrieval of various transmembrane receptors, we hypothesised that VPS35 may participate in the trafficking of ITGB3. A co-IP assay showed that VPS35 interacts with ITGB3 in GC cells (Fig. 6D). Immunofluorescence staining revealed a profound increase or decrease in cell surface ITGB3 levels after VPS35 overexpression or knockdown, *respectively*, in GC cells compared with control cells (Supplementary Fig. 6C). Using mass spectrometry analysis of MGC-803 cell and bioinformatic analysis, we identified that four post-translational modifications of VSP35, namely succinylation, methylation, phosphorylation and acetylation, among which phosphorylation was the most predominant (Fig. 6E). Subsequent analysis revealed that phosphorylation of VPS35 at S671 enhanced its interaction with ITGB3 at site R261 by site prediction (Fig. 6F). Co-Immunoprecipitation assay showed that a diminished binding of VPS35 to ITGB3 in the absence of a phosphorylase inhibitor. In contrast, this binding was preserved with the presence of the inhibitor (Fig. 6G). Take together, these results indicated that phosphorylation of VPS35 was enhanced in GC cells, and phosphorylated VPS35 has enhanced interaction with ITGB3. Furthermore, it was evident that ITGB3 recycling back to the cell surface following internalisation was increased and decreased in VPS35-overexpression and VPS35-knockdown cells, *respectively* (Fig. 6H, I). However, neither VPS35 overexpression nor silencing had a substantial effect on ITGB3 internalisation (Fig. 6H and Supplementary Fig. 6D).

RGD is an Arg-Gly-Asp peptide motif involved in binding and inhibiting integrin receptors. Notably, RGD treatment significantly reversed VPS35 overexpression-induced cell proliferation, migration and invasion (Supplementary Fig. 6E–G). Western blot analysis showed that RGD treatment increased the level of MST1/2, LATS1 and YAP phosphorylation but markedly reversed the elevated levels of FAK, SRC and AKT phosphorylation induced by VPS35 overexpression in tumour cells (Fig. 6J and Supplementary Fig. 6H). These results were further confirmed by the observation that RGD treatment reverses VPS35-induced YAP nuclear localisation (Fig. 6K, L and Supplementary Fig. 6I). Furthermore, positive correlations among VPS35, YAP and ITGB3 at the protein level were also demonstrated by western blotting of samples from 27 GC patients (Fig. 6M–P). These data indicate that the integrin-FAK-SRC axis mediates VPS35-induced YAP activation and GC cell progression.

### VPS35-dependent YAP activation Induces IL-6 in GC cells

**Fig. 7:**
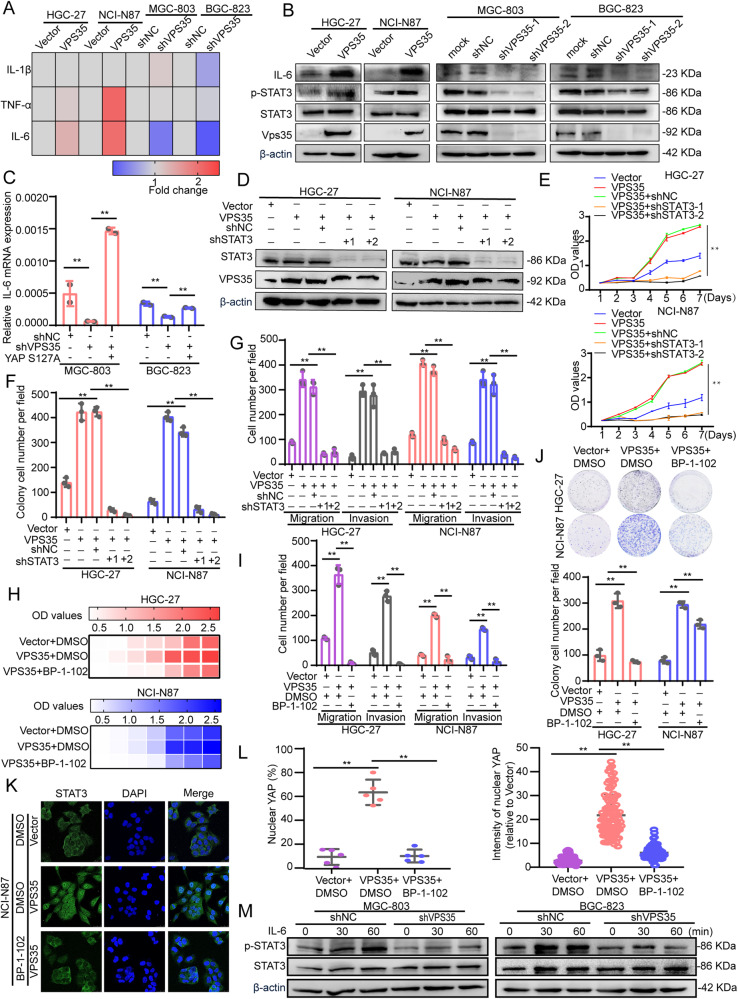
VPS35-dependent YAP activation induces IL-6 secretion in tumour cells. **A** Heatmap showing the expression of IL-6, IL-1β and TNF-α in VPS35-overexpressing GC cells and VPS35-knockdown GC cells. **B** The levels of IL-6, p-STAT3 and STAT3 in VPS35-overexpressing and VPS35-knockdown GC cells. **C** The expression of IL-6 was assessed by qRT‒PCR in MGC-803 or BGC-823 cells infected with shNC or shVPS35 and transfected with empty vector vs. vector expressing YAP^S127A^. **D** Western blot analysis detected the expression of STAT3 and VPS35 in VPS35-overexpressing cells treated with STAT3 shRNA. **E**, **F** CCK8 assays and colony formation assays were used to evaluate cell proliferation in VPS35-overexpressing cells treated with STAT3 shRNA. **G** Cell migration and invasion were analysed in VPS35-overexpressing cells treated with STAT3 shRNA. **H**–**I** CCK8 assays and colony formation assays were used to evaluate cell proliferation in VPS35-overexpressing cells treated with BP-1-102. **J** Cell migration and invasion were analysed in VPS35-overexpressing cells treated with BP-1-102. Immunofluorescence assay evaluating the nuclear translocation of STAT3 in VPS35-overexpressing NCI-N87 cells *with or* without BP-1-102 treatment (**K**). Quantification of the ratio and the intensity of nuclear STAT3 staining (**L**). **M** VPS35-knockdown GC cells were treated with IL-6 at different times as indicated, and the levels of p-STAT3 and STAT3 were determined by Western blotting. *p < 0.05, **p < 0.01.

Reportedly, YAP activation leads to the expression of proinflammatory genes such as IL-6, IL-8, and IL-1β [29]. Another recent report indicates that IL-6 is a target of YAP and plays an important role in maintaining the stemness of breast cancer cells [30]. Consistent with these findings, we found that overexpression of VPS35 increased the expression of IL-6 but not TNF-α and IL-1β in GC cells. Conversely, knockdown of VPS35 inhibited the expression of IL-6 in GC cells (Fig. 7A). Western blot analysis further confirmed that overexpression of VPS35 increased IL-6 expression and STAT3 phosphorylation in GC cells. Conversely, knockdown of VPS35 decreased IL-6 expression and STAT3 phosphorylation in GC cells (Fig. 7B). The VPS35 knockdown-induced decrease in IL-6 levels was reversed by the expression of YAP (S127A), a constitutively active form of YAP (Fig. 7C). Therefore, these results showed that VPS35-dependent YAP activation induced IL-6 expression in GC cells.

To confirm the role of IL-6/STAT3 in VPS35-induced GC proliferation, migration and invasion, STAT3 shRNA and a STAT3 inhibitor (BP-1-102) were used in VPS35 overexpressing GC cells. The results showed that VPS35 overexpression-induced cell proliferation, migration and invasion could be reversed by treatment with STAT3 shRNA and BP-1-102 (Fig. 7D–J and Supplementary Fig. 7A–C). The immunofluorescence assay results showed that BP-1-102 suppressed STAT3 nuclear translocation in VPS35-overexpressing GC cells (Fig. 7K, L). Furthermore, knockdown of VPS35 inhibited phosphorylated STAT3 in IL-6-induced cells (Fig. 7M). In conclusion, these results revealed that VPS35 promoted growth, migration and invasion via the IL-6/STAT3 pathway in a YAP-dependent manner in GC cells.

### STAT3 directly binds the VPS35 promoter and induces VPS35 expression in GC cells

The above results showed that VPS35 promoted cell proliferation, migration and invasion through the activation of STAT3 in GC cells. We wondered whether STAT3 could directly regulate VPS35 expression in GC cells.

**Fig. 8:**
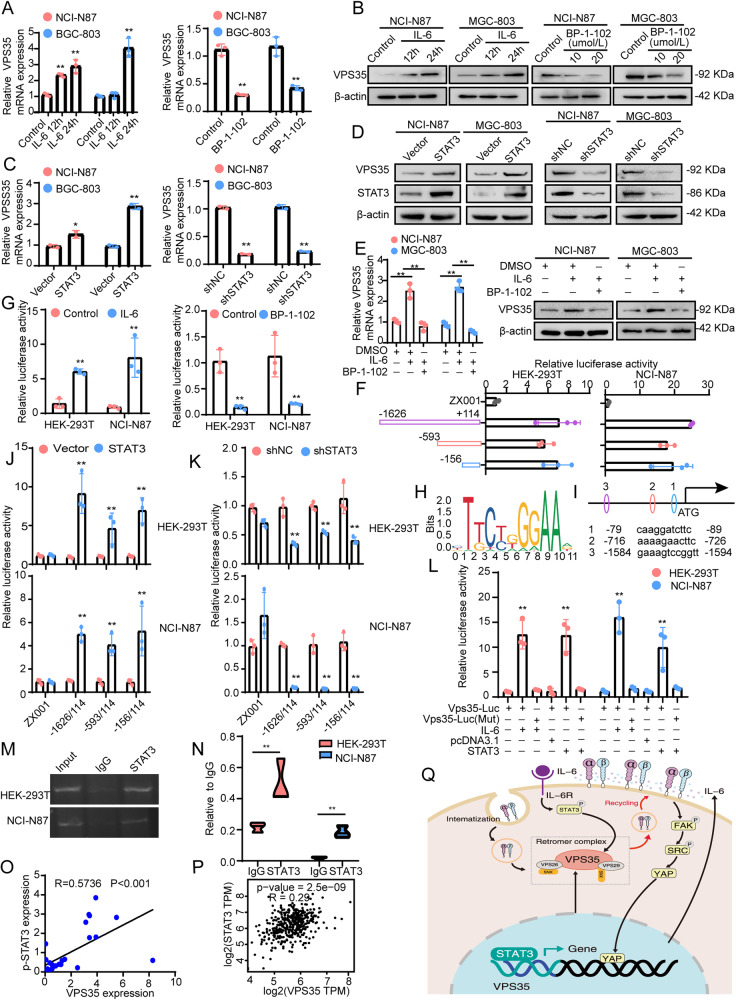
STAT3 directly binds the VPS35 promoter and induces VPS35 expression in GC. **A** The expression of VPS35 mRNA was assessed by qRT‒PCR in GC cells treated with IL-6 or BP-1-102. **B** The expression of VPS35 was assessed by Western blotting in GC cells treated with IL-6 or BP-1-102. **C** The expression of VPS35 mRNA was measured by qRT‒PCR in GC cells transfected with STAT3 plasmid or shRNA. **D** The expression of VPS35 was detected by Western blotting in GC cells transfected with STAT3 plasmid or shRNA. **E** The expression of VPS35 was assessed by qRT‒PCR and Western blotting in GC cells treated with BP-1-102 in the presence of IL-6. **F** HEK-293T and NCI-N87 cells were transfected with different luciferase reporter vectors containing truncation mutants of VPS35. The corresponding luciferase activities were determined by reporter gene assays. **G** HEK-293T and NCI-N87 cells were transfected with VPS35 luciferase reporter vectors (−1626/+114). Cells were treated with IL-6 or BP-1-102 for 24 h. Relative luciferase activities were determined by reporter gene assays. **H** STAT3 binding motif. **I** JASPAR analysis showed three potential STAT3-binding sites (scores > 8) within the promoter region of VPS35. **J** HEK-293T and NCI-N87 cells were transfected with different luciferase reporter vectors containing truncation mutants of VPS35 and STAT3 or vector. The corresponding relative luciferase activities were determined by reporter gene assays. **K** HEK-293T and NCI-N87 cells were transfected with different luciferase reporter vectors containing truncation mutants of VPS35 and shSTAT3 or vector. The corresponding relative luciferase activities were determined by reporter gene assays. **L** HEK-293T and NCI-N87 cells were transfected with VPS35 luciferase reporter vectors containing wild-type or mutant STAT3-binding sites, −156/+114. Cells were treated with IL-6 or transfected with a STAT3 vector or pcDNA3.1. The corresponding relative luciferase activities were determined by reporter gene assays. **M** Agarose electrophoresis for ChIP analysis of STAT3 binding to the VPS35 promoter. **N** qRT‒PCR for ChIP analysis of STAT3 binding to the VPS35 promoter. **O** The correlation between VPS35 and p-STAT3 protein levels in 42 GC tissues was analysed. **P** The correlation between the expression levels of VPS35 and STAT3 in GC tissues was analysed using TCGA datasets. **Q** Schematic representation of the VPS35/FAK-SRC-YAP/STAT3 positive feedback loop promoting GC progression. *p < 0.05, **p < 0.01.

First, we detected the expression of VPS35 in IL-6-treated GC cells. The results revealed that IL-6 induced VPS35 expression in a time-dependent manner (Fig. 8A, B). In contrast, BP-1-102 inhibited the expression of VPS35 (Fig. 8A, B). Similar results were also found in STAT3-overexpressing and STAT3-knockdown cells (Fig. 8C, D). Furthermore, our results showed that BP-1-102 treatment significantly reversed IL-6-induced VPS35 expression (Fig. 8E). Thus, these data suggested that STAT3 could regulate VPS35 expression in GC cells.

Next, we performed a reporter assay to identify whether the transcription factor STAT3 could control VPS35 transcription. The results showed that IL-6 stimulation increased VPS35 promoter activity in HEK-293T and NCI-N87 cells (Fig. 8G).

Considering that the VPS35 promoter contains 3 putative STAT3-binding sites (JASPAR 2022), we next defined which binding site(s) was/were responsive to STAT3 (Fig. 8H, I). To this end, serial truncations of the VPS35 promoter were created according to the location of the STAT3-binding sites (Fig. 8F). Luciferase assays showed that STAT3 overexpression or STAT3 knockdown increased or decreased VPS35 promoter activity, *respectively* (Fig. 8J, K). Therefore, we speculated that STAT3-binding sites may be located between nucleotides -156 and +114. Moreover, STAT3 did not increase the activity of the VPS35 promoter containing a mutated putative STAT3 binding site (site 1) (Fig. 8L). The ChIP assay data demonstrated that STAT3 directly bound to the VPS35 promoter (Fig. 8M, N). Western blot assays revealed a significant positive correlation between p-STAT3 and VPS35 protein levels in human GC tissues (Fig. 8O, *R* = 0.5736, *p* < 0.001). Furthermore, the TCGA GC cohort also revealed a significant positive correlation between the mRNA levels of STAT3 and VPS35 (Fig. 8P), suggesting that STAT3 could regulate VPS35 expression. Therefore, all of these results indicated that a positive feedback regulatory network involving VPS35 and STAT3 expression exists in GC.

### Knockdown of VPS35 sensitises GC cells to 5-FU and cisplatin

Considering the above results, we evaluated whether VPS35 is a potential target for GC therapy. Because fluorouracil (5-FU) and cisplatin are classical treatments for GC, we explored whether the knockdown of VPS35 enhances the effect of 5-FU and cisplatin in GC cells. CCK8 and colony formation assays showed that the combination of VPS35 knockdown and 5-FU or cisplatin treatment had stronger inhibitory effects on cell growth than either treatment alone (Supplementary Fig. 8A–D). Furthermore, VPS35 knockdown enhanced 5-FU- or cisplatin-induced cell apoptosis (Supplementary Fig. 8E–H). Therefore, these results suggested that VPS35 knockdown sensitises GC cells to 5-FU and cisplatin and may serve as a potential therapeutic strategy.

## Discussion

In the present study, we demonstrated that VPS35 was upregulated in GC and closely related to the prognosis of GC. Furthermore, VPS35 overexpression promoted the proliferation and metastasis of GC cells. Mechanistically, VPS35 activates FAK-SRC kinases through integrin-mediated outside-in signalling, which results in the activation of YAP and, subsequently, IL-6 expression in tumour cells. Interestingly, we found that STAT3 was also involved in the induction of VPS35 expression, pointing to the existence of a positive feedback loop in GC. Moreover, the knockdown of VPS35 sensitised GC cells to 5-FU and cisplatin.

The retromer is an evolutionarily conserved protein complex required for endosome-to-Golgi retrieval of receptors for lysosomal hydrolases. Accumulating evidence has demonstrated that VPS35 plays a critical role in Parkinson’s disease [31, 32]. However, research on the potential role of VPS35 in cancer is relatively limited. VPS35 regulates the sorting of FGFR3 and its trafficking to the plasma membrane and promoted the proliferation of HCC cells through the PI3K/AKT signalling pathway [8]. Consistently, another study demonstrated that the KLF7/VPS35 axis promotes HCC cell proliferation and invasion by activating Ccdc85c-medicated β-catenin pathway [33]. In breast cancer, VPS35 increases the proliferation and invasion abilities of cells and is essential for autophagy completion [9]. In this study, we found that the expression of VPS35 was upregulated and related to poor prognosis in GC, implying that it has a potential tumour oncogenic role in GC. Through gain-of-function and loss-of-function experiments, we demonstrated that VPS35 positively regulates the proliferation and peritoneal metastasis of GC cells.

Compelling evidence revealed that dysregulation of Hippo signalling contributes to the development of multiple cancers, including GC [34]. As a downstream effector, YAP plays a key role in the ability of the Hippo pathway to control cell proliferation; it has mostly been reported as an oncoprotein and its elevated expression and nuclear localisation have been frequently observed in human cancers [35, 36]. Therefore, the role of YAP as a promising and important therapeutic target has been increasingly recognised. However, compared to those in other tumour suppressors, mutations on Hippo components are less common [37]. Thus, alternative mechanisms may underlie Hippo inactivation during GC development, such as dephosphorylation of related kinases including MST1/2 and LATS1/2. Here, we found that VPS35 serves as a Hippo regulator to favour LATS1 dephosphorylation, thus impeding Hippo signalling. VPS35 overexpression significantly decreased LATS1 phosphorylation and enhanced YAP activation, whereas VPS35 knockdown restored LATS1 phosphorylation and prevented YAP nuclear translocation. Therefore, our findings indicate that VPS35 enhances tumour proliferation and metastasis by regulating YAP activation during gastric carcinogenesis.

Cancer cells can release many types of soluble factors to remodel tissue microenvironments to sustain their survival and growth [38]. Research has revealed an important role for the IL-6/STAT3 axis in FGF19-driven hepatocarcinogenesis while blocking this axis in hepatocytes abolishes FGF19-induced tumorigenesis [39]. Another report demonstrates that cancer-associated fibroblast-derived IL-6 increased c-Met expression in *MET*-unamplified GC cells through the IL-6/IL-6R/JAK2/STAT3 signalling pathway [40]. Here, we found that VPS35-induced YAP expression leads to an increase in IL-6 expression, while overexpressing YAP S127A is sufficient to restore the decreased levels of IL-6 induced by VPS35 knockdown. On the other hand, VPS35 overexpression increased the cell surface expression of ITGB3, which implies that VPS35 is essential for the sorting and trafficking of ITGB3 to the plasma membrane. Furthermore, we found that the levels of phosphorylated FAK and SRC were markedly increased or decreased following VPS35 overexpression or knockdown *respectively*. RGD has been shown to bind to the αvβ3 integrin that is expressed on the surface of angiogenic blood vessels or tumour cells, which is useful for the development of new therapeutic assessments and diagnostic and theragnostic application [41]. Notably, RGD treatment reversed the elevated levels of FAK, SRC and AKT phosphorylation, as well as YAP expression, induced by VPS35 in GC cells through disrupting integrin-mediated outside-in signalling. Using mass spectrometry analysis of MGC-803 cell and bioinformatic analysis, we identified phosphorylation of VPS35 was enhanced in GC cells, and phosphorylated VPS35 has enhanced interaction with ITGB3.Therefore, the integrin-FAK-SRC axis mediates YAP dependent IL-6/STAT3 signalling and is responsible for the VPS35-mediated promotion of GC proliferation and metastasis.

Accumulating evidence suggest that the Janus kinases (JAKs) and signal transducer and activator of transcription (STAT) proteins, particularly STAT3, are among the most promising new targets for the treatment of cancer [42]. In the tumour microenvironment, the IL-6/STAT3 axis drives the proliferation, invasiveness, and metastasis of cancer cells, and strongly suppresses the antitumour immune response [43]. IL-6/STAT3 also suppresses the antigen presentation ability of dendritic cells [44]. In addition, activated YAP interacts with STAT3 to induce the activation and nuclear translocation of STAT3, hence boosting the proliferation, migration and tube formation of HRMECs via VEGF signalling following hypoxia [45]. Thus, crosstalk between STAT3 and YAP activation may facilitate tumour progression. Here, we found that VPS35 induced YAP nuclear translocation and resulted in IL-6/STAT3 pathway activation. Surprisingly, STAT3 can directly bind to the VPS35 promoter and promote VPS35 expression. Thus, a positive feedback loop between VPS35 and STAT3 controls GC progression. Our work also showed that the STAT3 inhibitor BP-1-102 or shSTAT3 not only suppressed VPS35 expression but also hindered VPS35-mediated cell proliferation and invasion in GC cells. Therefore, inhibition of STAT3 could be a promising therapeutic strategy for GC. Some STAT3 inhibitors, such as napabucasin and BBI608, have been proven to suppresse tumour progression and enhance antitumour effects in human and mouse models [46, 47]. Furthermore, we also verified that VPS35 knockdown sensitises GC cells to the 5-FU and cisplatin, suggesting that the inhibition of VPS35 in combination with 5-FU and cisplatin may represent a potential strategy for treating GC.

In conclusion, we demonstrate that VPS35 enhances the activation of YAP via the integrin-FAK-SRC pathway, thereby increasing the expression of IL-6 in GC cells. STAT3 can directly bind to the VPS35 promoter and promote VPS35 expression (Fig. 8Q). Our study provides strong evidence to support the hypothesis that the VPS35/YAP/IL-6 loop bridges the interaction between tumour cells during gastric malignant progression. Taken together, our results show that targeting VPS35- and IL-6/STAT3-mediated tumour interactions may be an attractive therapeutic strategy for GC.

## Materials and methods

### Bioinformatics analysis

In this study, we used the GEPIA database (http://gepia.cancer-pku.cn) to analyse the expression levels of VPS35, CEACAM6, CLDN4, CXCL1, EREG, HOXA13, LIF, MMP12 and ONECUT2 in TCGA-STAD and their relationships with the prognosis and survival of patients. The RNA-sequencing data and clinical information of GC patients were downloaded from the TCGA database (https://portal.gdc.cancer.gov) or the GEO database (https://www.ncbi.nlm.nih.gov/geo/). The TCGA-STAD, GEO63228, GEO36968 and GEO66229 datasets were used to analyse the expression and prognosis of VPS35 in Asian and non-Asian GC patients. GEO63228, GEO193453, GEO172032 and GEO224056 were used to find differentially expressed genes in GC and adjacent normal tissues. The differentially expressed genes (DEGs) between tumour and nontumour samples were identified by using the DEseq package in R and filtered according to the threshold | log2 [fold change (FC)]| ≥ 1 and adjusted *p* < 0.05. Then, an online Venn diagram website(http://bioinformatics.psb.ugent.be/webtools/Venn/) was used to identify the intersecting genes.

### Cell lines and cell culture

GC cell lines (NCI-N87 and AGS) and HEK 293T cells were purchased from the American Type Culture Collection (ATCC, Manassas, VA, USA), and other GC cell lines (MKN-45, SGC-7901, HGC-27, MGC-803 and BGC-823) and GES-1 cells were obtained from the Chinese Academy of Science. All cell lines were authenticated by short tandem repeat (STR) profiling and tested to ensure that they were mycoplasma-free. All cells were cultured in DMEM supplemented with 10% foetal bovine serum (FBS), 100 U/ml penicillin G and 100 μg/ml streptomycin sulphate; incubated at 37 °C in a humidified atmosphere with 5% CO_2_; and used within 6 months of resuscitation.

### Plasmids, lentivirus production and cell transduction

The pLVX-VPS35 plasmid and the VPS35 lentiviral shRNA plasmid were supplied by GeneCopoeia (Guangzhou, China). The YAP lentiviral shRNA plasmid was cloned into the pLKO.1 vector. The human YAP ORF was cloned into the pcDNA3.1 vector to generate the pcDNA3.1-YAP S127A plasmid, whose encoded protein cannot be phosphorylated by LATS kinases and is thus in the cell nucleus. The STAT3 lentiviral shRNA plasmid was supplied by GeneChem (Shanghai, China). The target sequences are listed in Supplementary Table 1. For lentivirus production, HEK-293T cells were transfected with the corresponding vector with the packaging plasmid psPAX2 and the envelope plasmid pMD2.G (Addgene) using Polyplus (jetPRIME) according to the manufacturer’s instructions. The viruses were harvested 48–72 h after transfection, and GC cells were infected with 1 × 10^6^ recombinant lentivirus-transducing units in the presence of 6 μg/ml polybrene (Sigma).

### Immunohistochemistry (IHC)

A tissue microarray containing 89 GC tissues was used for immunohistochemical procedures. Briefly, sections were deparaffinized with xylene and rehydrated before antigen retrieval by heating to just below the boiling temperature in Tris/EDTA buffer (pH 9.0) for 20 min in a microwave oven. The primary antibodies used for the immunohistochemical assay were specific for VPS35, Ki67, and YAP. The proportion of positively stained cells was scored based on the following percentages of cells with positive staining: 0, 0%; 1, <10%; 2, 10–35%; 3, 35–75%; and 4, and >75%. These results were scored on a scale of 0 to 4 by two independent investigators. The individual scores for the staining intensity and percentage of positive cells were then multiplied to calculate the immunoreactivity score for each sample. Samples with a final staining score of ≤4 were considered to exhibit low expression, and those with a score of >4 were considered to exhibit high expression. Information on the antibodies is provided in Supplementary Table S3. The study was approved by the Ethics Committee of Ruijin Hospital, Shanghai Jiao Tong University School of Medicine.

### RNA extraction and quantitative real-time RT-PCR (qRT-PCR)

RNA was extracted using the TRIzol reagent method. Complementary DNA (cDNA) was obtained via reverse transcription of equal amounts of RNA according to the protocols for the PrimeScript™ RT Reagent Kit with gDNA Eraser (Perfect Real Time). QRT-PCR analyses were performed using an ABI Prism 7500 System (Applied Biosystems, Carlsbad, CA, USA) with SYBR^®^ *Premix Ex Taq* (Takara, Dalian, China). The threshold cycle value of the target gene was normalised to that of GAPDH, and the relative expression of the target genes was calculated using the 2^−ΔΔCt^ method. The primer sequences are listed in Supplementary Table 2.

### Western blotting

Total proteins extracted from the cells using RIPA lysis buffer (Millipore) were separated on 10% SDS‒PAGE gels (EpiZyme) and transferred onto polyvinylidene fluoride membranes using a wet blotting system (BIO-RAD). The membranes were blocked with 5% BSA in TBS-T, incubated with primary antibodies overnight at 4 °C, probed with HRP-conjugated secondary antibodies for 2 h at room temperature, and visualised using enhanced chemiluminescence reagent (Pierce, Rockford, IL, USA). Information on the antibodies used is provided in Supplementary Table S3.

### Cell proliferation and colony formation assays

For cell proliferation, cells were seeded in 96-well plates overnight and estimated by the Cell Counting Kit-8 (CCK8) (Bimake, USA). The absorbance of the wells was measured at 450 nm. For colony formation assays, cells were plated in six-well plates and incubated at 37 °C in a humidified atmosphere with 5% CO_2_ for 1–2 weeks. Colonies were fixed with 4% phosphate-buffered formalin (pH 7.4) for 30 min and stained with Giemsa. Each experiment was performed in triplicate.

### Transwell assay

Cells were seeded in the upper chamber of a transwell (8 μm pore size) or in a Matrigel-coated transwell (BD Biosciences, NJ) in serum-free medium. The lower chamber contained DMEM with 10% foetal bovine serum as a chemoattractant. After 12 h of incubation, the nonmigrated or noninvaded cells were gently removed from the upper chamber using a cotton swab. The cells were fixed and stained using crystal violet staining solution and counted in five randomly chosen visual fields.

### Flow cytometry analysis

For cell cycle analysis, cells were plated in six-well plates overnight and then synchronised by adding culture medium containing 2 mM thymidine or 0.3 µM nocodazole. The cells were trypsinized at the indicated time, washed twice with cold PBS and fixed with 70% ethanol at −20 °C overnight. The cells were then washed twice with PBS and incubated with 10 mg/ml RNase A, 400 mg/ml propidium iodide and 0.1% Triton X in PBS at 4 °C for 30 min. Finally, the cells were analysed by flow cytometry. Cell cycle profile distributions were determined with Modfit LT 3.2 software.

For apoptosis analysis, 1 × 10^6^ cells were plated in six-well plates and cultured overnight. The chemotherapy drugs 5-FU and cisplatin were then added for 24 h, and the cells were trypsinized and stained with 7-AAD and APC-Annexin V (BD Biosciences Corporation) according to the manufacturer’s protocol. Finally, the cells were analysed by flow cytometry within one hour. FlowJo VX software was used to analyse the data.

### Immunofluorescence confocal imaging

GC cells were plated on Lab-Tek chamber slides (Nunc), fixed with 4% paraformaldehyde for 30 min at room temperature, and permeabilized with 0.1% Triton X-100 in PBS for 15 min. The slides were incubated with primary antibodies in blocking solution overnight at 4 °C in a humidified chamber. Subsequently, the glass slides were washed three times in PBS and incubated with Alexa Fluor 546-conjugated or Alexa Fluor 488-conjugated secondary antibodies and 4′,6-diamidino-2-phenylindole in blocking solution for 30 min at 37 °C. Images were obtained with a Leica TCS SP8 confocal system (Leica, Microsystems). Information on the antibodies used is provided in Supplementary Table S3.

### Nuclear and cytoplasm extraction

Nuclear and cytoplasmic extraction assays were conducted using a nuclear protein extraction kit (Abmart) according to the manufacturer’s protocol. Briefly, the cells were washed with ice-cold PBS and then resuspended in buffer A (protease inhibitor). After gentle vortexing at 4 °C for 10 min, the cells were centrifuged at 14,000 rpm and 4 °C for 10 min. The resultant supernatant was then kept as the cytosolic fraction. The pellet was then resuspended in buffer C (protease inhibitor). After gentle vortexing at 4 °C for 30 min, the cells were centrifuged at 14,000 rpm and 4 °C for 10 min. The pellet was then resuspended in buffer D (protease inhibitor). After ten rounds of ultrasonic treatment and vortexing at 4 °C for 30 min, the cells were centrifuged at 14,000 rpm at 4 °C for 10 min. The resultant supernatants were then kept as the nuclear fraction. Both the nuclear and cytosolic fractions were quantified using the BCA method, and proteins were analysed by SDS‒PAGE and Western blotting. Histone 3.1 and GAPDH were used as markers for the nucleus and cytoplasm, respectively.

### Internalisation and recycling assays

Internalisation and recycling assays were conducted as reported previously [16]. Briefly, cell-surface proteins were labelled with 0.05 mg/ml cleavable EZ-LinkTM Sulfo-NHS-SS-Biotinin in cold PBS for 30 min at 4 °C. Then, the cells were washed three times in PBS containing 100 mM glycine. Prewarmed serum-free DMEM was added to the cells, and the cells were allowed to internalise biotin-labelled surface proteins at 37 °C for the indicated times. Internalisation was stopped by transferring the cells to 4 °C. Surface biotin was removed by washing three times for 10 min in cold stripping buffer (50 mM L-GSH reduced, 75 mM NaCl, 75 mM NaOH, and 1% bovine serum albumin (BSPS, and 10 mM EDTA, pH 8.0). For recycling assays, cells were allowed to internalise biotin-labelled surface proteins at 37 °C for 30 min. Surface biotin was removed as described above. Following treatment with stripping buffer, the cells were washed once in cold PBS. Prewarmed serum-free growth medium was added, and the cells were incubated at 37 °C for the indicated times. Cell lysates were incubated with streptavidin-conjugated agarose beads for 2 h at 4 °C. The beads were washed three times in RIPA buffer with a phosphorylase inhibitor and examined by Western blot analysis.

### Co-immunoprecipitation (Co-IP) assay

Briefly, cells were harvested in RIPA lysis buffer with a phosphorylase inhibitor, incubated for 30 min on ice and centrifuged at 12,000 × *g* for 10 min. The protein A/G agarose beads were incubated with antibodies against ITGB3 and VPS35 or negative control IgG overnight at 4 °C with rotation. The complexes were washed with elution buffer and boiled for 10 min, followed by Western blot detection.

### Luciferase assay

The VPS35 promoter and 5′-truncated sequences of the VPS35 promoter were supplied by IGEbio (Guangzhou, China). The VPS35 promoter (bp −1626/+114 relative to the ATG start codon) was cloned into the luciferase reporter gene vector zx001. Two 5′-truncated sequences of the VPS35 promoter were generated by PCR and cloned into the luciferase reporter gene vector zx001. The fidelity of the constructs was confirmed by sequencing. The primer sequences are listed in Supplementary Table S2.

Cells were grown in 24-well culture plates and cotransfected with mixtures of the corresponding reporter plasmid and the indicated plasmids in each experiment according to the standard protocol. Luciferase activity was detected using a Dual-Luciferase Report Assay (Promega) system in accordance with the manufacturer’s instructions.

### Chromatin immunoprecipitation (ChIP) assay

ChIP assays were conducted using the EZ-ChIP Kit (Millipore) according to the manufacturer’s protocol. Briefly, GC cells were grown to 90% confluence, and crosslinking was performed with 1% formaldehyde for 10 min before quenching with 1 × glycine. The cell lysates were sonicated to shear the DNA to sizes of 500–1000 bp. A rabbit anti-STAT3 antibody or rabbit IgG was used to immunoprecipitate DNA-containing complexes. After reversing cross-linking of protein/DNA complexes to free DNA, qRT‒PCR was used to detect the STAT3-binding site in the VPS35 promoter region. The primer sequences are listed in Supplementary Table 2.

### In vivo growth and metastasis assays

For in vivo growth, a total of 1 × 10^6^ cells (NCI-N87 pLVX or NCI-N87 VPS35/MGC-803 shNC or MGC-803 shVPS35) suspended in 200 μl serum-free DMEM were injected into the left axillary fossa of nude mice. For the peritoneal metastatic xenograft model, NCI-N87 pLVX or NCI-N87 VPS35 cells were lentivirally transduced with the firefly luciferase (FFLuc) fusion vector (Genepharma) and selected with 2 μg/ml puromycin (Gibco). Then, 2 × 10^6^ GC cells were trypsinized, suspended in 200 μl serum-free DMEM and injected into the abdomen of mice. Four weeks later, the tumour mass and distribution in the abdomen were assessed by bioluminescence imaging. Sample size calculation was performed using IndiGo software, and subsequently, peritoneal metastases were evaluated by H&E staining. All of the experiments were approved by the Shanghai Medical Experimental Animal Care Commission.

### Statistical analysis

All data are presented as the mean ± SD. Statistical comparisons of data were performed using a two-tailed Student’s *t* test or one-way ANOVA for multiple comparisons. The *χ*^2^ test was used to analyse the relationships between the expression of VPS35 and clinicopathologic parameters. Overall survival curves were calculated using the Kaplan–Meier method and compared using the log-rank test. Univariate and multivariate analyses were performed using Cox regression analysis. Correlations were evaluated by using a Pearson correlation test. *p* < 0.05 was considered to indicate statistical significance. (*p < 0.05, **p < 0.01). Statistical analyses were performed using SPSS 24.0 software.

### Supplementary information

Supplementary information

## Data Availability

All data generated or analysed during this study are included in this paper.
