# Supplementary material for: VPS35 promotes gastric cancer progression through integrin/FAK/SRC signalling-mediated IL-6/STAT3 pathway activation in a YAP-dependent manner
Source: Oncogene. 2023 Nov 11;43(2):106–22. doi: 10.1038/s41388-023-02885-2 (PMC10774127; doi:10.1038/s41388-023-02885-2)
Supplement: Supplementary file 1 — Supplementary information [file 41388_2023_2885_MOESM1_ESM.docx]

#### Supplementary Tables and Figures

#### Supplementary Table 1. The sequences of shRNA target

| Identifier | Forward(5’-3’) |
| --- | --- |
| VPS35-1 | CCGGACTTCTATGTTATCACC |
| VPS35-2 | GGTCTGTTTCTTCGAAATTAC |
| YAP-1 | CCCAGTTAAATGTTCACCAAT |
| YAP-2 | GCCACCAAGCTAGATAAAGAA |
| STAT3-1  STAT3-2 | GCACAATCTACGAAGAATCAA  CTCAGAGGATCCCGGAAATTT |

**Supplementary Table 2.** The sequences of gene-specific primers used for qRT-PCR, vector constructs and ChIP assay

| Gene name | Forward(5’-3’) | | | Reverse (5’-3’) |
| --- | --- | --- | --- | --- |
| **Primers for qRT-PCR** | | | |  |
| VPS35 | | CTCAAGACCAGGTGGATTCC | | TGGCAGTGTGAAGCGAATC |
| IL-1β | | GGCCCTAAACAGATGAAGTGC | | CCAGCATCTTCCTCAGCTTG |
| IL-6 | | CTTCTCCACAAGCGCCTTCG | | TTCTCAGGGCTGAGATGCCG |
| TNF-α | | CCCAGGGACCTCTCTCTAATCA | | AGCTGCCCCTCAGCTTGAG |
| GAPDH | | AGAAGGCTGGGGCTCATTTG | | AGGGGCCATCCACAGTCTT C |
| **Primers for vector constructs** | | | |  |
| VPS35(-1626/+114) | | CTAGTACGCGTATTTAAATGTCGACGTGCTCAAATGCTTTCAGGC | | AGCATCGGCCATGGTGGCGGATCCCTGAGGGGACTGCTGTGTTGTAGGCATGGCG |
| VPS35(-593/+114) | | ACATTTCTCTACTAGTACGCGTATTTAAATGTCGACAAGCCCAGCCCTGGTCTTGCGCG | | CTTAATGTTCTTAGCATCGGCCATGGTGGCGGATCCCTGAGGGGACTGCTG |
| VPS35(-156/+114) | | ACATTTCTCTACTAGTACGCGTATTTAAATGTCGACTCGCACGCTGTGGTGAGATTGGC | | CTTAATGTTCTTAGCATCGGCCATGGTGGCGGATCCCTGAGGGGACTGCTG |
| promoter mut (-156/+114) | | CGCTCAACTTTGGCAAGCTTGGTACCGAGCTCG | | CTATAGAATAGGGCCCTCTAGATGCATGCTC |
| **Primers for ChIP** | | | |  |
| VPS35 promoter for STAT3 | | | TGAGATTGGCTGGCGCTCA | TAGCCTCCCGCGGTCATGT |

**Supplementary Table 3.** Antibodies used in this study

| Antibody | catalog | Dilution | Company |
| --- | --- | --- | --- |
| **For Western blotting** | | | |
| VPS35  PCNA  p-YAP(ser127)  YAP  p-LAST1  LAST1  p-MST1/2  MST1  p-FAK  FAK  p-Src  Src  p-AKT  AKT  ITGB3  P-Rb (Ser807/811)  Rb  Cyclin D1  CDK6  CDK4 | T58074  ab29  CST-13619  CST-14074  TA8163  TA7669  CST-49332  CST-3682  CST-8556  CST-71433  CST-12432  CST-2109  CST-4060  CST-4691  T55158  CST-8516  sc-102  ab226977  sc-7961  sc-56277 | 1:500  1:800  1:500  1:500  1:500  1:500  1:500  1:500  1:500  1:500  1:500  1:500  1:500  1:500  1:500  1:800  1:300  1:500  1:300  1:300 | Abmart Abcam  CST  CST  Abmart  Abmart  CST  CST  CST  CST  CST  CST  CST  CST  Abmart  CST  Santa Cruz  Abcam  Santa Cruz  Santa Cruz |
| MMP9  MMP2  MMP7  p-STAT3 | TA5228  T57164  TA0218  CST-9145 | 1:500  1:500  1:500  1:500 | Abmart  Abmart  Abmart  CST |
| STAT3  Histone H3.1  GAPDH | CST-12640  P30266  10494-1-AP | 1:500  1:5000  1:5000 | CST  Abmart  Proteintech |
| β-actin | A3854 | 1:10000 | Sigma |
| Secondary antibody | HRP conjugated goat anti-rabbit IgG | 1:3000 | Sigma |
| Secondary antibody | HRP conjugated goat anti-mouse IgG | 1:3000 | Sigma |
| **For Immunohistochemistry** | | | |
| VPS35 | T58074 | 1:50 | Abmart |
| Ki67  YAP | GT209407  CST-14074 | 1:50  1:50 | Genetech  CST |
| Secondary antibody | Envision kit (HRP, rabbit/mouse, DAB+) | Ready-to-use | DAKO |
| **For Immunofluorescence staining** | | | |
| YAP  ITGB3 | 13584-1-AP  T55237 | 1:60  1:50 | Proteintech  Abmart |
| STAT3 | CST-12640 | 1:60 | CST |
| Secondary antibody | Alexa Fluor 546 anti-rabbit IgG | 1:50 | Invitrogen |
| Secondary antibody | Alexa Fluor 488 anti-mous IgG | 1:50 | Invitrogen |

**Supplementary Figures**


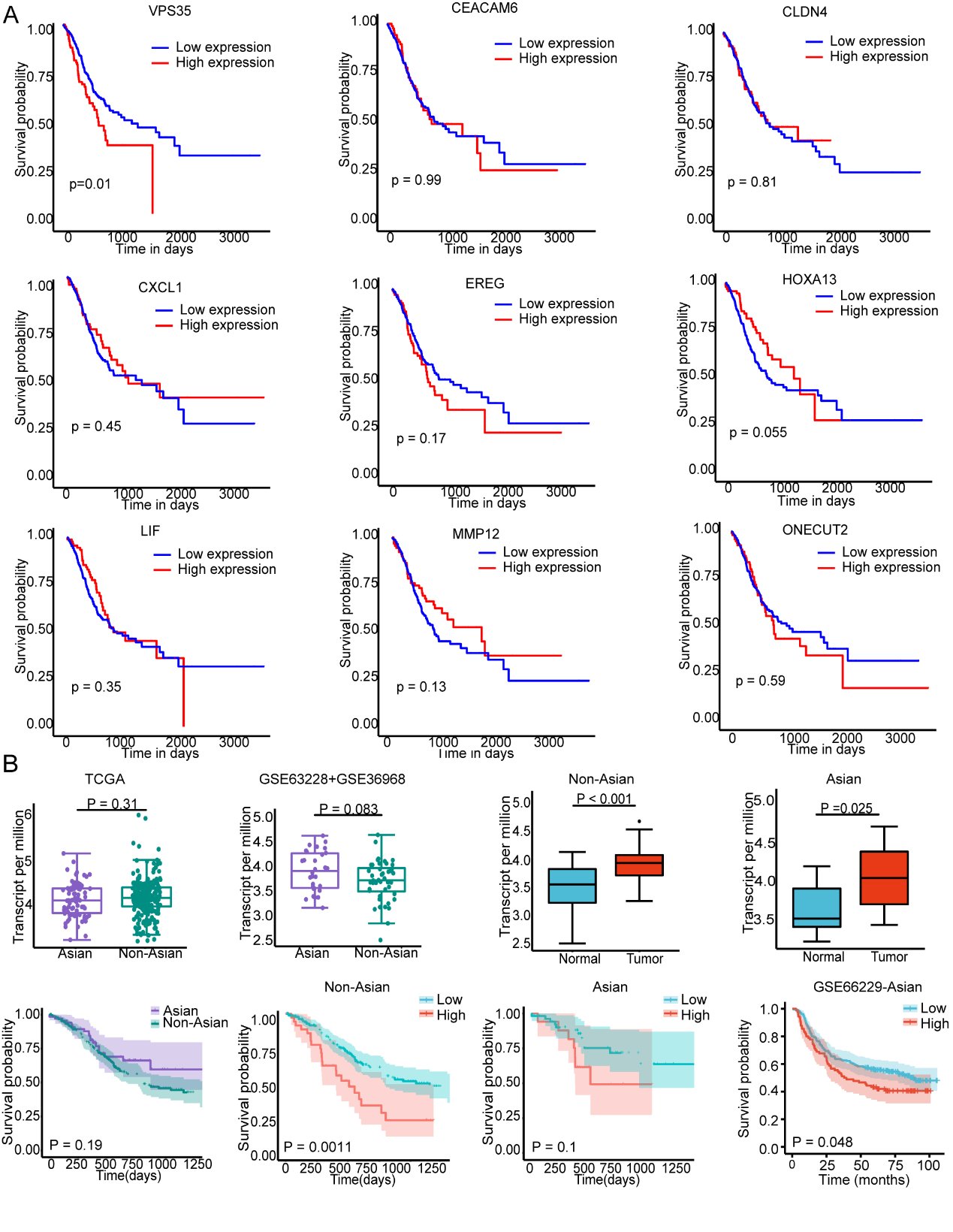


**Figure S1.** (A) Kaplan–Meier survival curves of patients with GC according to VPS35, CEACAM6, CLDN4, CXCL1, EREG, HOXA13, LIF, MMP12 and ONECUT2 expression using data sets from TCGA. (B) The expression of VPS35 in Asian GC patients compared with Non- Asian GC patients, or the expression of VPS35 in GC tissues compared with corresponding noncancerous gastric tissues in Asian GC patients or Non- Asian GC patients was analyzed using data sets from TCGA, GSE63228 or GSE36968.Kaplan–Meier survival curves of patients with GC in Asian versus Non-Asian, in Asian or in Non-Asian according to VPS35 expression using data sets from TCGA or GSE66229. **p* < 0.05; ***p* < 0.01.

**
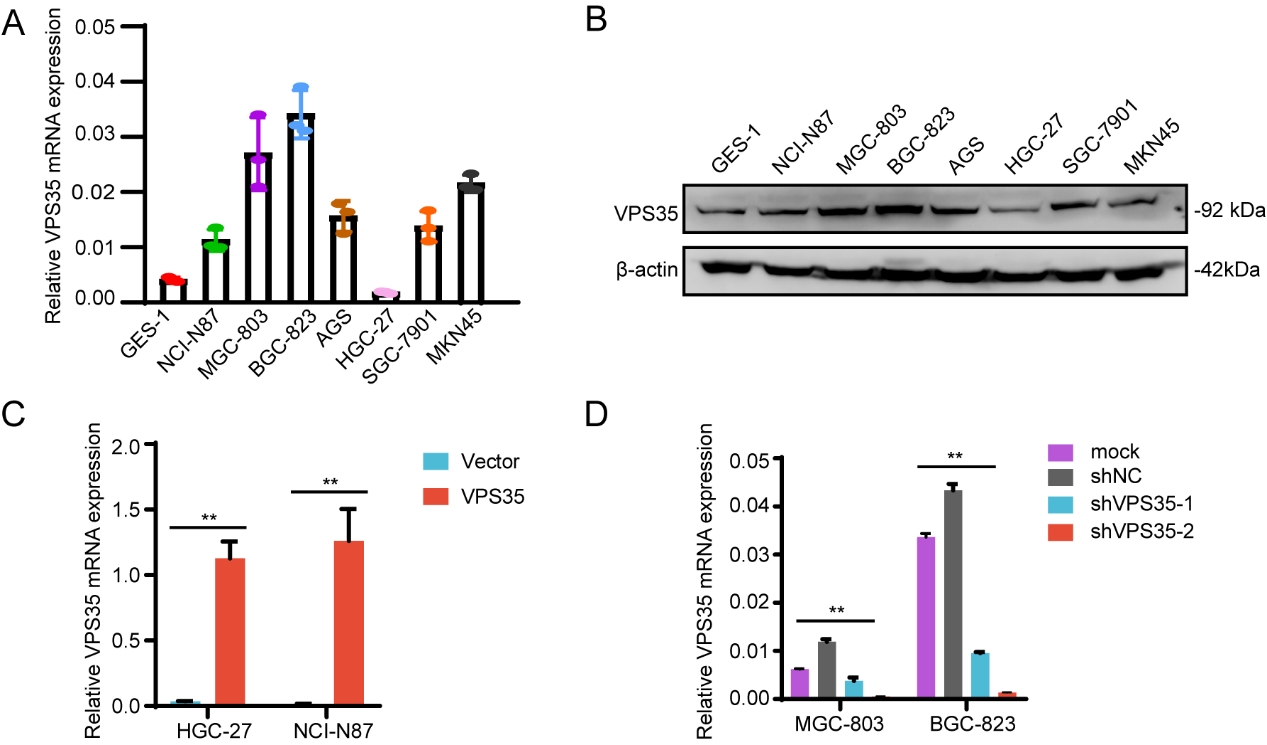
**

**Figure S2.** (A) The expression of VPS35 in GC cell lines was detected by qRT-PCR. (B) The expression of VPS35 was determined by western blotting in GC cells. (C-D) The expression of VPS35 was determined by qRT-PCR in GC cells with VPS35-overexpressing(C) and VPS35 knockdown(D). **p* < 0.05; ***p* < 0.01.


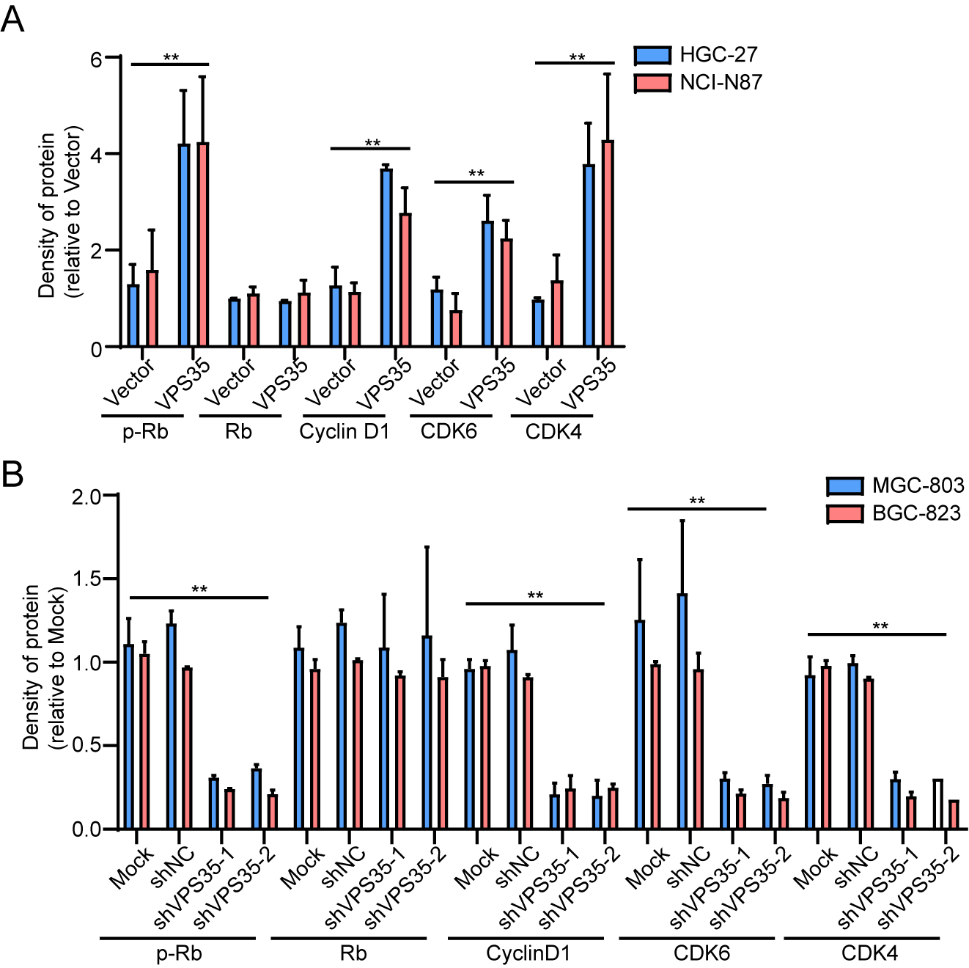


**Figure S3.** Density of Figure 3E and 3F. (A) Density of Figure 3E in the text. (B) Density of Figure 3F in the text. **p* < 0.05; ***p* < 0.01.


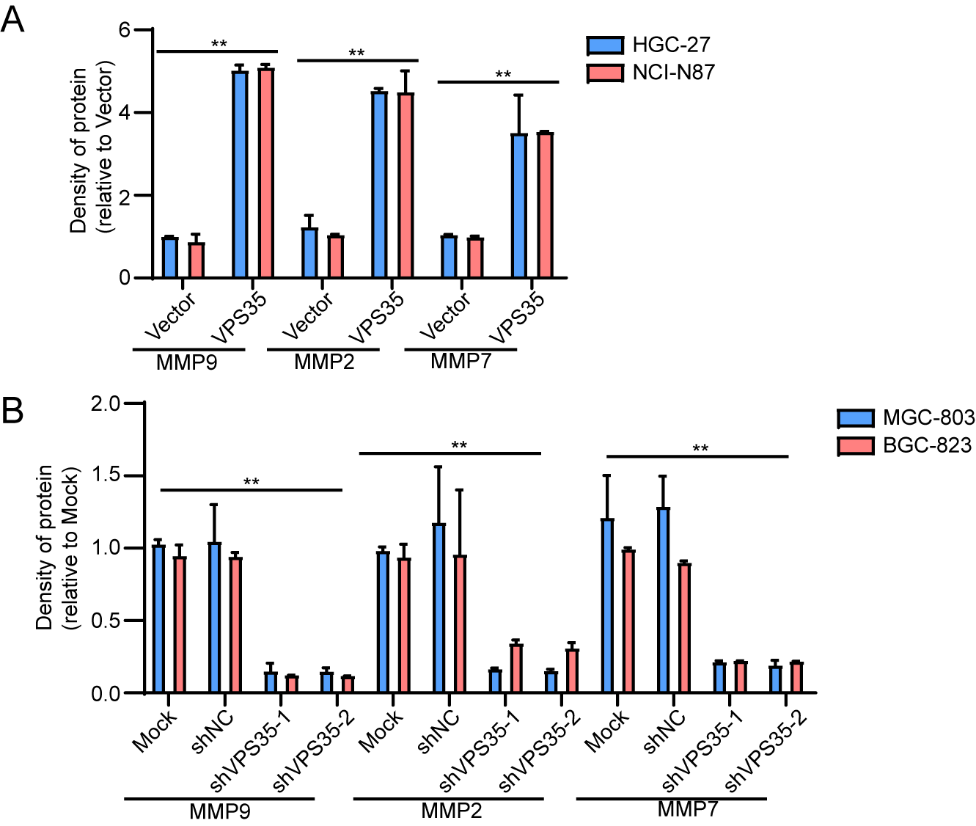


**Figure S4.** Density of Figure 4C and 4D. (A) Density of Figure 4C in the text. (B) Density of Figure 4D in the text. **p* < 0.05; ***p* < 0.01.


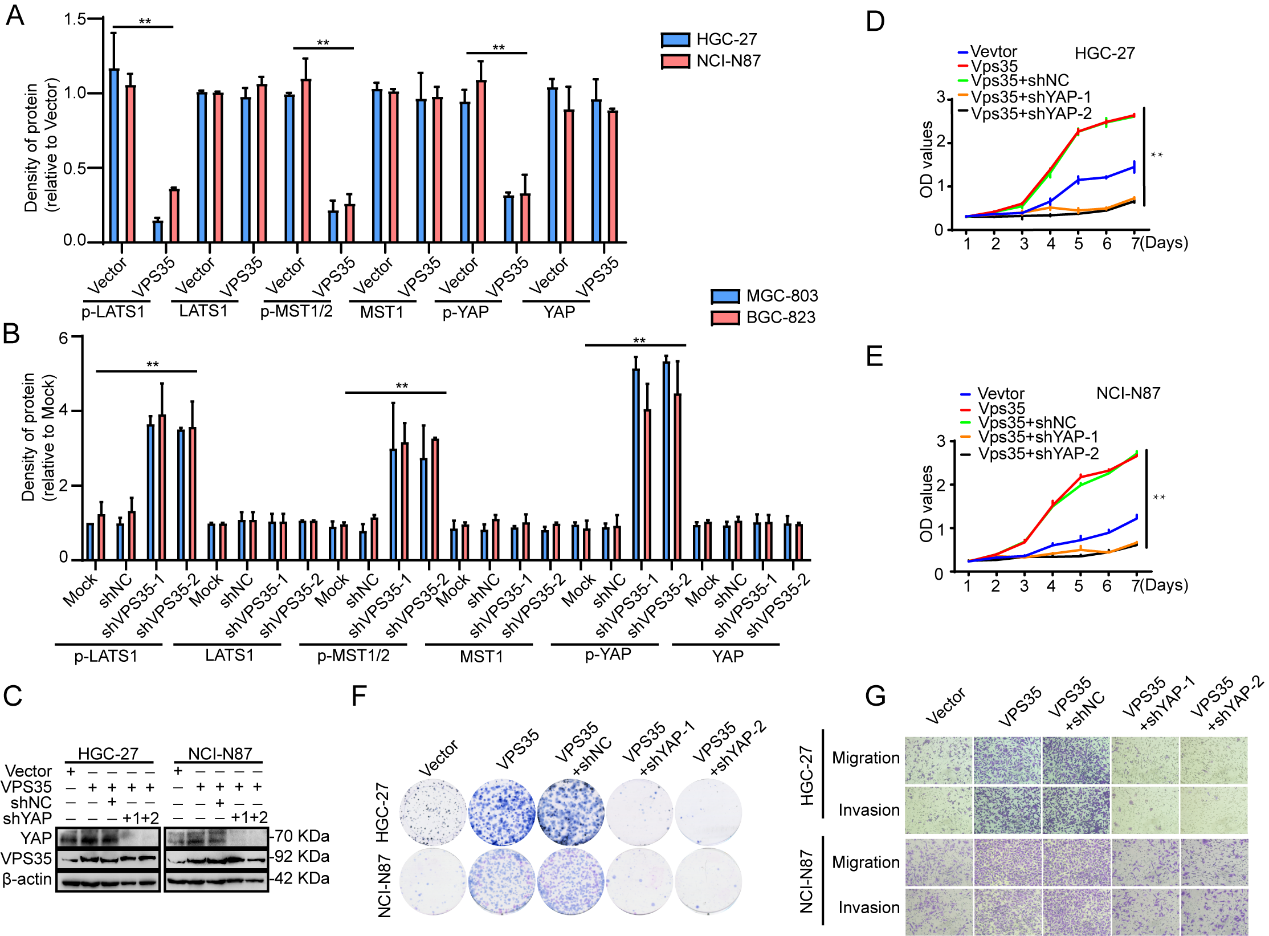
**Figure S5.** (A) Density of Figure 5C in the text. (B) Density of Figure 5D in the text. (C) The expression of VPS35 and YAP was analyzed in VPS35 overexpressing GC cells treated with YAP shRNA. (D-E) Cell proliferation was analyzed in VPS35-overexpressing cells transfected with YAP shRNA. (F) Colony formation assay was analyzed in VPS35 overexpressing cells treated with YAP shRNA. (G) Cell migration and invasion were analyzed in VPS35 overexpressing cells treated with YAP shRNA. **p* < 0.05; ***p* < 0.01.


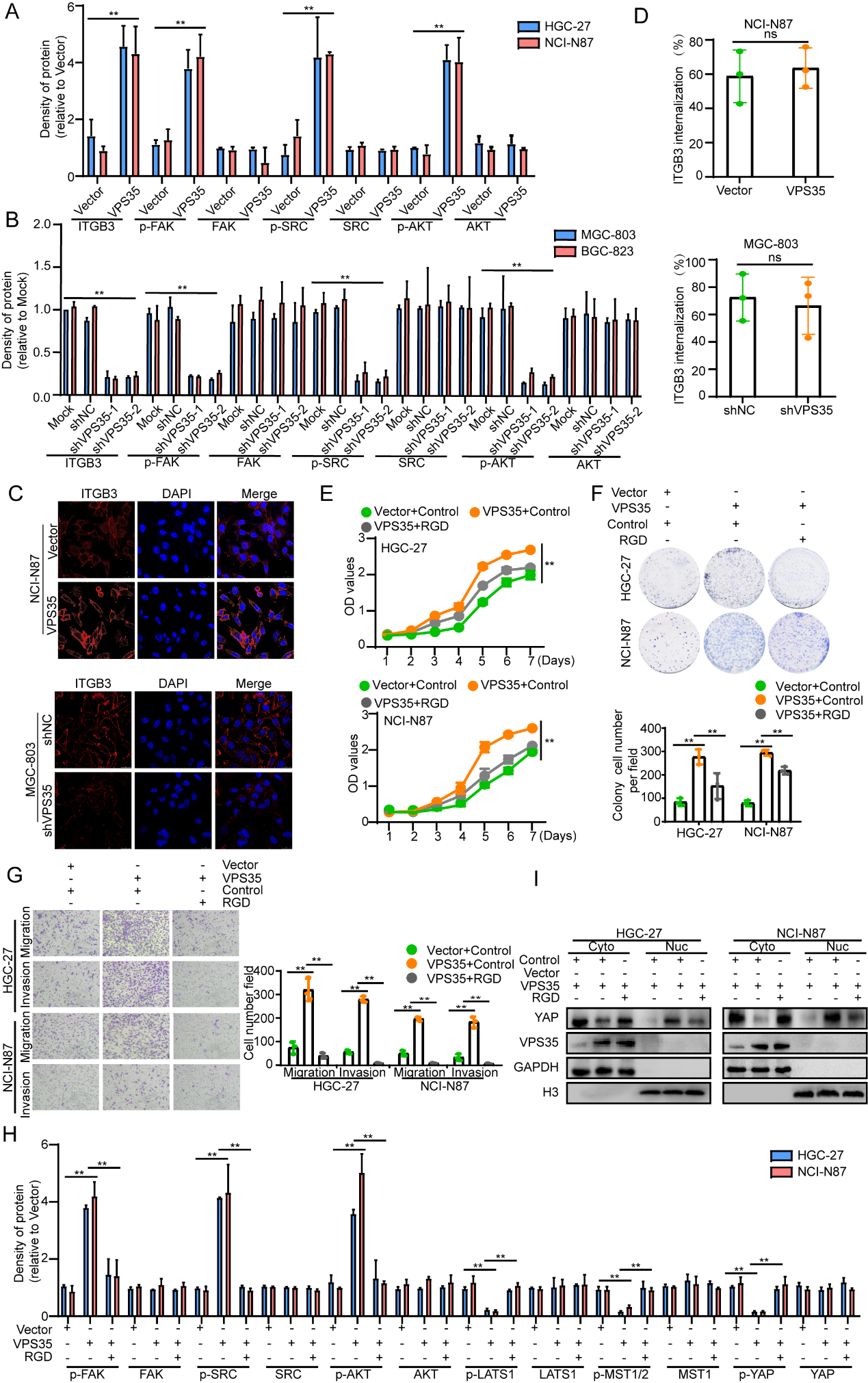
**Figure S6.** (A) Density of Figure 6B in the text. (B) Density of Figure 6C in the text. (C) Immunofluorescence assays detected the protein expression levels of ITGB3 and VPS35 in NCI-N87 and MGC-803 cells. (D) Quantification of internalized ITGB3 in VPS35 overexpressing or knockdown cells and their corresponding control cells, respectively. (E) VPS35-overexpressing GC cells were treated with RGD as indicated, and cell growth was evaluated by CCK8 assay. (F) VPS35-overexpressing GC cells were treated with RGD as indicated, and cell growth was evaluated by colony formation assay. (G) Cell migration and invasion were analyzed in VPS35-overexpressing cells treated with RGD. (H) Density of Figure 6J in the text. (I) Nucleocytoplasmic separation and western blot analysis of YAP cellular localization change in VPS35-overexpressing cells treated with RGD. **p* < 0.05; ***p* < 0.01.


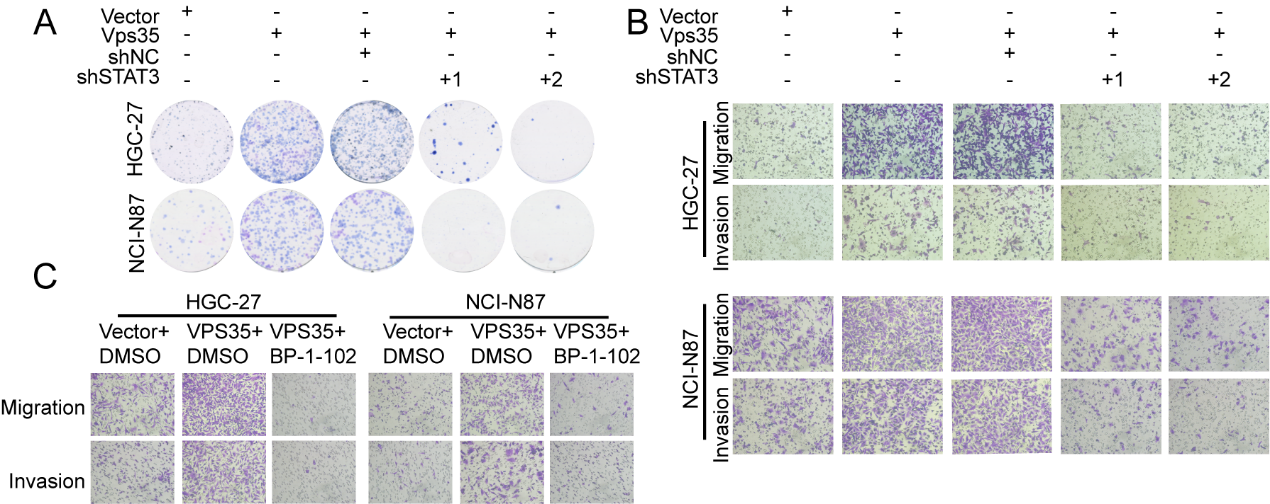


**Figure S7.** (A) Cell proliferation was analyzed by a colony-formation assay in VPS35-overexpressing cells treated with YAP shRNA. (B) Cell migration and invasion were analyzed in VPS35-overexpressing cells treated with YAP shRNA. (C) Cell migration and invasion were analyzed in VPS35 overexpressing cells treated with BP-1-102.


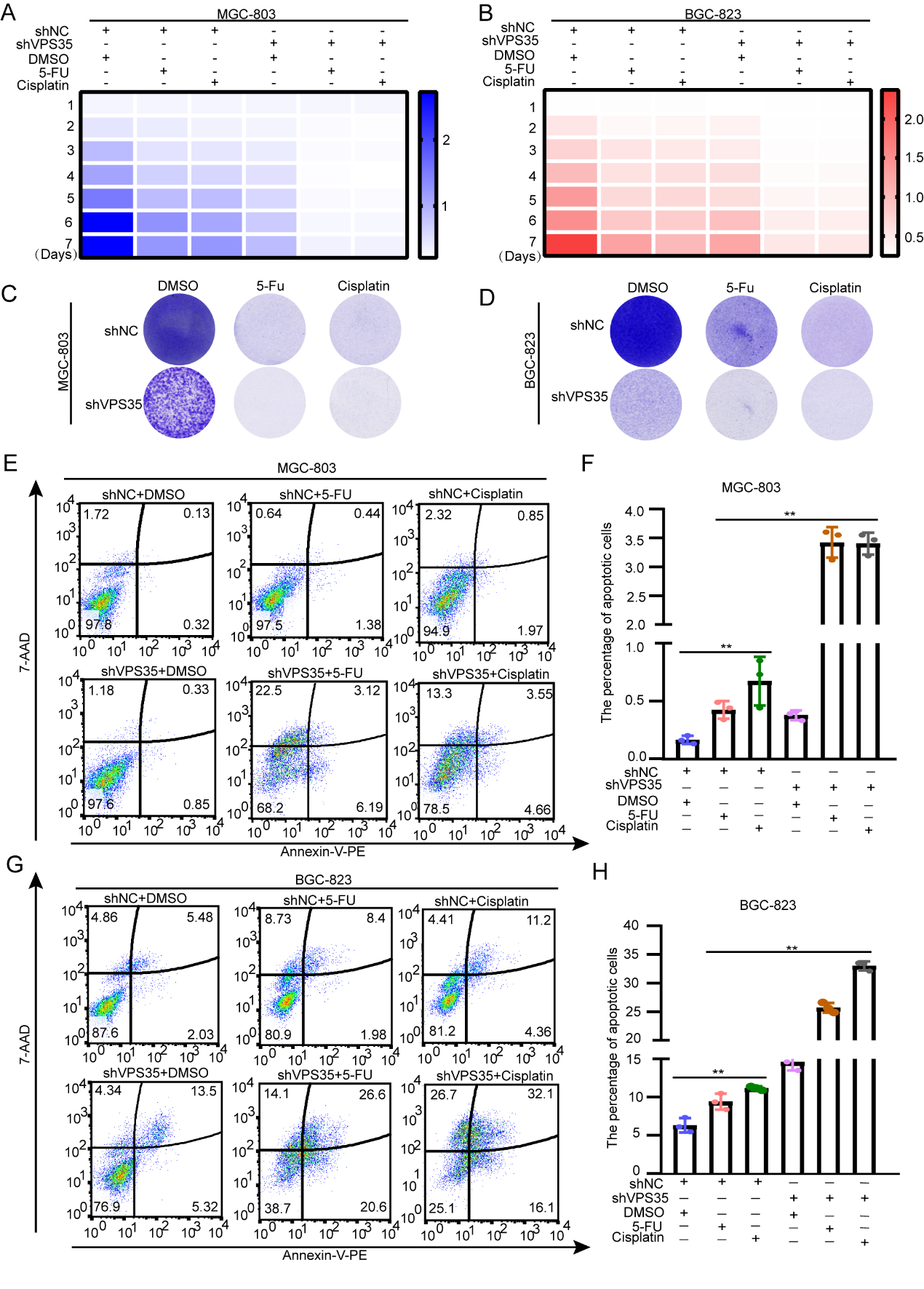


**Figure S8.** (A-B) Heatmap demonstrated cell proliferation in VPS35 knockdown MGC-803 and BGC-823 cells treated with 5-FU(5ug/ml) or Cisplatin(2ug/ml). (C-D) Cell proliferation was assessed by a colony formation assay in VPS35 knockdown MGC-803 and BGC-823 cells treated with 5-FU(5ug/ml) or Cisplatin(2ug/ml). (E-H) VPS35 knockdown MGC-803 and BGC-823 cells and corresponding control cells were treated with 5-FU(5ug/ml) or Cisplatin(2ug/ml) for 24 h, and apoptotic cells were assessed by flow cytometry. *p < 0.05; **p < 0.01.
